# The impact of periodic updates to health benefits plan: access gains without cost savings?

**DOI:** 10.1007/s10754-025-09394-7

**Published:** 2025-04-09

**Authors:** Oscar Espinosa, Paul Rodríguez-Lesmes, Jhonathan Rodríguez, Diego Ávila, Sergio Basto, Giancarlo Romano, Lorena Mesa, Hernán Enríquez

**Affiliations:** 1 Directorate of Analytical, Economic and Actuarial Studies in Health, Instituto de Evaluación Tecnológica en Salud (IETS), Bogotá, D.C., Colombia; 2 Economic Models and Quantitative Methods Research Group, Centro de Investigaciones para el Desarrollo, Universidad Nacional de Colombia, Bogotá, D.C., Colombia; 3 School of Economics, Universidad del Rosario, Bogotá, D.C., Colombia

**Keywords:** Health benefit plans, Universal health coverage, Health technology assessment, Health expenditures, Cost-containment policies, Pharmaceutical markets, Colombia, Health policy, I13, I18, H51, C23

## Abstract

Expanding explicit Health Benefit Plans (HBP) is a key strategy for achieving universal health coverage while maintaining financial sustainability. However, little is known about the broader effects of periodic updates to these plans on healthcare utilization, expenditures, and market dynamics. This study examines the impact of including new health technologies in Colombia’s HBP covered by the Capitation Payment Unit (CPU) between 2012 and 2019, using administrative data and a difference-in-differences approach with multiple periods. Our results indicate that inclusion in the HBP-CPU led to a substantial increase in utilization and access, particularly in remote areas, but had mixed effects on expenditures. While the number of unique users and prescription frequency rose significantly, the cost per user remained stable for procedures but increased for medications, likely due to higher demand and market structures. These findings suggest that although periodic HBP updates enhance access and effective coverage, they do not necessarily generate cost savings. Strengthening health technology assessment processes, integrating price regulation policies, and implementing cost-containment mechanisms are essential for ensuring the financial sustainability of health systems that regularly update benefit plans.

**Supplementary Information:**

The online version contains supplementary material available at 10.1007/s10754-025-09394-7.

## Introduction

Achieving universal health coverage (UHC) is the pursuit of a comprehensive set of quality health technologies (medications, procedures, etc.) that all individuals can access regardless of their circumstances (named Health Benefits Plans, HBP) (Glassman et al., 2017; Cotlear et al., 2015). Coverage has three essential dimensions: who is covered by insurance, what services are included in the insurance plan, and what proportion is financed by people - named out-of-pocket health expenditure - (Etienne et al., 2010; Schreyögg et al., 2005). Currently, several countries are achieving nearly universal coverage with high levels of financial protection (Lozano et al., 2020; McKee et al., 2013). However, there are countries with similar levels of health expenditures but with vast differences in adequate access to health services (Lozano et al., 2020).

The challenge of implementing HBP lies in the financing, which involves defining financial sources and establishing consistent budgets with public (or private) resources. Currently, no society can afford everything. Both in the US and Europe, there is a growing concern about the prices of new technologies, particularly pharmaceutical products, which account for more than 16% of the health budget in the European Union (Conti et al., 2021; OECD, 2018). Most of the efforts in containing health expenditures are centred on supply-based regulations such as price or margin controls (Von der Schulenburg et al., 2011).

On the demand side, the objective is to convince (or force) prescribers and patients to use specific products or procedures. While an explicit HBP (positive list of covered technologies) could potentially restrain the supply of certain technologies, it also has a demand-based component by indicating to prescribers evidence of a product’s usage for treating specific conditions. Still, defining and sustaining an explicit plan is arduous in practice. Barriers include legal provisions, political pressures from interest groups, financial pressures, and the technical and administrative capacity to update it (Glassman et al. 2017). This work aims to understand how updating HBP (inclusions and exclusions of health technologies every certain time) affects the usage, expenditures, and market conditions of health technologies. We do so for the case of Colombia, a country that has been using this strategy since the early 90s. Our estimates are based on the inclusion of 260 health procedures and 354 medications between 2012 and 2019, that were used in the health system by 2012 but were not part of the explicit HBP list.

Like several middle-income countries, Colombia provides health insurance coverage to nearly all its citizens with one of the lowest out-of-pocket expenditures of the OECD and Latin America (Herrero & Herrera, 2018; Espinosa et al., 2024). However, it still has several shortcomings in terms of the quality and timeliness of health services (OECD, 2018). The institutional context of the country provides a special setting for the assessment of the inclusion of health technologies: in practice, the health system combines mandatory health insurance that contains an explicit HBP -that is financed through a capitation mechanism (henceforth, HBP-CPU)-1 with a process of periodic updating, with an implicit rationing scheme (technologies funded by specific case analysis, without an explicit list) in which some technologies that are not covered by the HBP-CPU are financed. Therefore, it is possible to observe how the use and expenditure on specific health technologies are financed before and after their inclusion in the explicit PBS-CPU (i.e., more expeditious access without administrative obstacles).

Most of the literature related to HBP studies the introduction of HBP on populations without prior access to health insurance, not the marginal expansion of health technologies in health coverage (Khan et al., 2020; Erlangga, 2018; El Omari & Karasneh, 2021; Green et al., 2021; Thuong, 2020). With this paper, we want to contribute to the literature that explores the implications of the role of coverage of health technologies on crucial aspects of health systems performance. In the following section, we describe the background of the Colombian HBP-CPU, its updating process, and the hypotheses linked to the inclusion of a health technology into the HBP-UPC. Next, datasets are presented, as well as details of econometric strategies. Finally, we present the discussion and conclusions following the results.

## Background: the Colombian HBP-CPU

Colombian HBP was set by Law 100 of 1993, which stated the basis of the UHC of the country. This law defined the managed competition between health insurers (Giedion & Villar, 2009; Escobar et al., 2009). People employed in the formal sector contribute to the payroll that allows them to belong to the contributory scheme (as well as their beneficiaries); people in the informal sector or who are not able to contribute, have access to health insurance through the subsidized scheme, which covers health services through public subsidies. Nowadays, Colombian HBP has effective coverage for technologies and health services that is not far from the United Kingdom system, similar to Chile, and substantially better than the Mexican (Lozano et al., 2020). Supplement A compares the HBP of Colombia with other countries, as well as their HBP updating process.

In Colombia, technologies are automatically financed by mandatory health insurance if they are listed in the explicit HBP-CPU list. However, health technologies not included in the HBP-CPU could be prescribed by physicians if they consider them valuable for their patients (unless the technology is explicitly excluded). Further details on the development of this system are presented in Supplement B. But how different was the prescription of technology inside and outside the HBP-CPU? What are the potential trade-offs for the patient and the physician to choose between an included technology A and a non-included technology B?

For physicians, the prescription of technology B involved substantial red tape. For the patient, it was mainly about the delay in receiving treatment and its implications in terms of health deterioration. Patients could also consider purchasing B out-of-pocket, but this was uncommon as most excluded technologies are expensive. Physicians usually belong to a provider institution with a contractual agreement with an insurance company. Therefore, the physician needed to request their company access to the technology, which might involve a meeting with a team to assess the requirement. The provider then needed to request the insurance company to authorise the usage of the technology, as it might not be recognised as it could be considered outside the contract. Internally, the insurance company had to decide on the authorisation, which involved meetings within them and with the providers’ physicians. The basis is that the insurer would have to claim these resources from the government outside the capitation contract -a procedure known as *recobro*-. Once the provider receives the authorisation, the company will purchase the technology if unavailable. This process could take weeks or months and might end up in the judiciary system. With the implementation of MIPRES in 2017, the administrative procedure was simplified. However, in some cases, the final payment for the technology to the provider could take months as it was considered an item outside the original contract between the provider and the insurer. Hence, provider institutions pressure their physicians to avoid using such technologies.

In the general scenario, unless the physician’s perceived benefit for using B over A is substantial, the balance would go for A. Hence, we hypothesise a substantial increase in the usage of technology B in the health system once included. The inclusion of B also means that it will be part of the information systems of insurers and providers, prompting an information treatment on those physicians who would not have considered option B otherwise. We expect this effect to be larger for products for which there is better information about their advantages in terms of efficiency and cost-efficiency. This is likely to be the case for those technologies for which there is a health technology assessment (HTA).

The next consideration is in terms of the economic consequences of the inclusion. In principle, higher demand for a product should reduce its price. Yet, this might depend crucially on the type of technology. Medications can be distributed through existent logistic systems without additional costs, but some procedures might need special equipment and specialised medical teams. As a result, we hypothesise lower expenditures per patient for medications but higher for procedures.

## Methods

### Data

We consider data between 2012 and 2019 from contributory scheme insurance companies that approve the validation system of the Ministry of Health and Social Protection (MHSP). Our dataset considers each health technology, its frequency of usage, its number of unique users, its expenditures, and the geographic area where the health technology was delivered to the patient. We use two databases called Suficiencia -technologies financed with the HBP-CPU- and Recobros -technologies not financed with the HBP-CPU-.

Two sets of exercises are considered: (i) For all technologies that were used at least once between 2012 and 2019, we study the extent of usage in terms of the number of users and their location; (ii) for technologies that had at least one user in all years between 2012 and 2019, we explore the frequency of use and expenditure per user.

We explore 9,118 technologies: 1,706 medicines and 7,412 procedures. From those, 58.6% had at least one user between 2012 and 2019 (747 medications and 4,603 procedures). Hence, it is important to highlight that for the second set of outcomes, our results are local for those already available and used in the market. This selection means that our analysis for those outcomes corresponds to the inclusion into the HBP-CPU of drugs and procedures available for several years in the market.

#### HBP-CPU updates

The administrative records list 702 technologies included in the HBP-CPU. After restricting the data to those used at least once between 2012 and 2019, 614 technologies remain, 260 procedures and 354 medications, representing 87.5% of the original list. When considering the set of technologies used all years from 2012 to 2019, we are left with 230 inclusions, comprising 162 medications and 68 procedures.

**Table 1:** Mean of products’ characteristics per year, for all non-HBP technologies by 2012

	2012	**2013**	**2014**		2015	2016		2017		2018		2019	
	**All**	**All**	**All**	New inclusions	All	All	New inclusions	All	New inclusions	All	New inclusions	All	New inclusions
	**(1)**	**(2)**	**(3)**	(4)	(5)	(6)	(7)	(8)	(9)	(10)	(11)	(12)	(13)
*Panel A. Any technology*													
Number of procedures	1,706	1,706	1,706	29	1,706	1,706	113	1,706	4	1,706	60	1,706	54
Number of medications	7,412	7,412	7,412	119	7,412	7,412	171	7,412	3	7,412	20	7,412	41
At least one user	0.759	0.748	0.737	1	0.756	0.821	1	0.822	1	0.787	1	0.811	1
Frequency per user	1.017	0.992	0.979	1.331	0.994	1.151	1.756	1.181	1.214	1.084	2.479	1.125	2.359
Unique users per million	669.26	740.116	840.172	1,273.84	797.371	1,236.80	3,831.02	1,257.05	169.307	1,200.63	101.122	1,311.38	531.158
Expenditure per user (million COP)	2.151	1.855	1.379	2.863	1.749	2.045	2.035	2.696	0.681	1.732	10.08	1.878	2.587
Expenditure per user (USD, 2019)	655.51	565.402	420.317	872.496	532.978	623.423	620.285	821.653	207.422	527.743	3,072.31	572.531	788.393
Technology is a procedure	0.813	0.813	0.813	0.804	0.813	0.813	0.602	0.813	0.429	0.813	0.25	0.813	0.432
Included in the HBP-CPU	0	0	0.022	1	0.025	0.063	1	0.067	0.857	0.082	1	0.094	1
Usage in scattered areas	0.373	0.382	0.386	0.236	0.41	0.47	0.535	0.467	0.571	0.456	0.563	0.485	0.684
HTA in previous years	0	0	0.004	0.169	0.005	0.01	0.025	0.01	0	0.021	0.55	0.025	0.242
*Panel B. Only medications*													
WHO essential medicine 2021	0.216	0.214	0.195	0.172	0.201	0.216	0.321	0.213	0	0.213	0.25	0.215	0.057
Price cap regulation	0.034	0.063	0.094	0.552	0.096	0.091	0.107	0.091	0	0.141	0.283	0.21	0.358
Only one firm per ATC5	0.036	0.029	0.03	0.041	0.035	0.043	0.049	0.043	0.286	0.041	0.287	0.042	0.126
Years from registry oldest product	12.972	13.85	14.25	14.414	14.72	15.41	18.857	15.96	16	16.63	15.067	17.435	15.113
Presence of generics	0.468	0.48	0.46	0.586	0.47	0.47	0.679	0.47	0.5	0.46	0.4	0.46	0.434
IHH of both market ATC4		2,966.85	2,919.92	1,693.34	2,428.76	2,601.79	2,529.89	2,436.34	4,915.35	2,939.78	3,470.86	2,952.22	2,340.44
IHH of both market ATC5		4,708.72	4,497.49	3,852.00	4,069.87	4,246.31	3,742.91	4,170.33	5,334.95	4,427.87	4,512.13	4,421.82	4,323.46
Firms per ATC4		56.001	54.669	67.241	55.691	55.672	77.661	56.964	9.5	57.242	51.717	55.8	59.792
Firms per ATC5		17.014	15.949	18.483	16.349	15.956	26.384	16.074	5.5	16.231	14.117	15.939	10.679
Share institutional market ATC4		0.334	0.45	0.623	0.615	0.632	0.739	0.63	0.386	0.483	0.495	0.503	0.713
Share institutional market ATC5		0.382	0.465	0.677	0.572	0.604	0.744	0.605	0.481	0.519	0.56	0.544	0.679

*Notes:* panel A presents usage variables derived from Suficiencia and Recobros datasets, plus the cumulative proportion of technologies included in the HBP-CPU, and with a HTA. Panel B is based on its own calculations using SISMED’s transactions per product for the previous two years, and INVIMA registries. HTA corresponds to the inclusion of health technologies that had an HTA study. *New inclusions* columns correspond to technologies included in the HBP-CPU in the year expressed in the respective column

The first two rows of Table 1 present the number of observations considered in our study (already included or not in the HBP-CPU). It shows the number of all procedures and technologies per year, and for the years in which there was an update, the number of the included technologies. Inclusions occurred mostly in 2016, followed by 2014. During the study period, most of the procedures included corresponded to diagnostic tests for neoplastic diseases, and gastro-oesophageal diseases, among others. In Supplement F we present a general idea of their clinical domains and four specific examples of technologies that we analyse in detail.

#### Variables of interest

We use two data sources (Suficiencia and Recobros) to obtain information about the use of specific technologies and the total expenditures on each, our main dependent variables (see Table 2).

**Table 2:** Definition of the variables of interest

**Variable**	**Definition**
At least one user	A dichotomous variable takes the value of 1 if there is at least one user of the technology in the country that year
Number of unique users per million affiliates	Total patients that have a record of usage of technology *i* divided by the number of insured persons in each year (in millions).
Frequency of usage	Total units delivered of technology *i* divided by the number of unique users to whom the technology *i* was delivered.
Expenditures per user	The total amount of money of technology *i* (in USD, 2019=100, the exchange rate of 3,281 COP per USD), divided by the total number of users.
Usage in scattered areas	A dichotomous variable takes the value of 1 if the technology was used in a special or remote area and takes the value of 0 otherwise (see Figure C1). There, transport costs and times are notoriously higher. In addition, there is usually a relative scarcity of supply health services available.

#### Other variables

HTA is designed to be used as a tool to consider the inclusion of technologies into insurance plans, national HBP, or private companies. We constructed an indicator variable to determine whether the technology was considered in any HTA study available each year. Market characteristics are also important in determining the expansion of a technology. For medications, we use administrative records of whole-seller-level transactions in the market originated by the pharmaceutical company or the importing firm. For medications, we calculate several variables based on both the ATC level four (ATC4) and level five (ATC5). Computed variables are a dummy variable that identifies if there are generics available in the market, the number of firms with sales in the market, a binary variable that indicates if there is only one firm with registries (a monopoly), the number of years since the register of the product, the industrial concentration via the Herfindahl-Hirschman Index (IHH), if there are products in the ATC5 subject to a price cap regulation, and the proportion of the market that is sold to the mandatory insurance companies or directly to health providers (institutional market). As many of these products’ transactions are reported to be traded only once per year, we consider a two-year window for them.

### Empirical strategy

According to what was commented previously, we considered health technologies exclusively not included in the HBP-CPU at the beginning of our study period (year 2012). There is a staggered adoption of treatment: inclusion occurs on different periods, and once a health service is included, it will remain *treated* forever.^2^

#### Probability of inclusion

To obtain an unbiased estimate of the impact of the inclusion into the HBP-CPU, we should compare technologies with similar growth potential if not included in the HBP. If this is not the case, and included technologies were expected to grow faster regardless of their inclusion, our estimates would be biased upwards. Hence, the first step is to understand empirically the selection process of those technologies eventually included. We explore which characteristics are linked to the probability of inclusion. We model the probability of inclusion in a given period *t* as follows:

1 $$\begin{aligned} Pr(G_{i,t}=1 | G_{i,t-1}=0 , X_{i,t-1} ) = \Lambda ( X'\beta ) \quad , \quad \end{aligned}$$

where $\Lambda (\cdot )$ is the logistic function, and $X_{i,t-1}$ corresponds to a set of control variables in the previous year. On top of the type of technology, it includes (i) the usage outcomes: number of unique users per million affiliates (i.e. enrollees) and expenditures per user. Also, (ii) a dummy that indicates if technology usage was considered in an HTA. And for medications only, (iii) market characteristics considering the last two years (for both ATC4 and ATC5): if there are generics available in the market, the number of firms with sales in the market, if there is only one firm with registries, the number of years since the register of the product, the industrial concentration via the IHH index, and the proportion of the market that is sold to the mandatory insurance companies.

#### Estimating the impact of a technology inclusion

The CS-DiD estimator applies when there is a staggered treatment assignment (Callaway & Sant’Anna, 2020).^3^ CS-DiD model ensures that each treated technology’s comparison group consists of only units that have not been treated yet. As with the standard DiD, the central assumption is the conditional parallel trends, but specific for the Not-Yet-Treated groups

The parameters of interest represent the average treatment effect on the treated (ATT) at period *t* for technologies included in year $g\in \mathcal {G}$ (the set of periods where there is an inclusion):

$$\begin{aligned} ATT(g,t) = E [ Y_{i,t}(g) - Y_{i,t}(0) | G_{i,g}=1 ] \quad , \quad for \quad t\ge g \quad , \end{aligned}$$

where $Y_{i,t}$ is one of the three outcome variables for technology *i* out of *J* technologies in year *t*. $Y_{i,t}(g)$ corresponds to its value if the technology started to be treated in period *g* (a particular normative resolution), and $Y_{i,t}(0)$ its counterfactual value in case of no treatment. $G_{i,g}$ takes the value of 1 if the technology *i* is included in the HBP in year *g*, and 0 otherwise (treatment start-time dummies). Then, we compute i) a general result (group time average), which averages across all periods and technologies; and ii) an event-study aggregation (dynamic effects) for each period after the inclusion into the HBP-CPU. We complement this analysis with a synthetic control strategy as a robustness check (Cavallo et al., 2010; Abadie et al., 2010; Abadie & Gardeazabal, 2003; Abadie, 2021). With this strategy, we ensure usage characteristics pre-intervention are similar, not only in their trends but also in the level.

## Results

### Inclusion of technologies, market conditions, and HTA heterogeneity

Table 3 presents the means by year of the outcome variables, the proportion of the technologies that are procedures (81.3%), the proportion included in the HBP-CPU by a given year (8.6% of the considered technologies by 2019), and the proportion which undergo an HTA. We observe an important increase in the mean of the usage variables between 2015 and 2016 and the expenditure variable only in 2017.

**Table 3:** Odds-Ratio after a logistic regression over technologies not included before in the HBP

	(1)	(2)	(3)	(4)	(5)
	All	Procedures	Medications		
Medication $$\times$$ unique users per 100K	0.763$$^{***}$$				
	(0.0690)				
Procedures $\times$ unique users per 100K	0.992				
	(0.0162)				
Unique users per 100K		1.001	0.701$$^{***}$$	0.628$$^{***}$$	0.615$$^{***}$$
		(0.00717)	(0.0768)	(0.0811)	(0.0816)
Medication $\times$ expenditure per user (1000 USD)	0.994				
	(0.0154)				
Procedures $$\times$$ expenditure per user (1000 USD)	1.024$$^{**}$$				
	(0.0105)				
Expenditure per user (1000 USD)		1.026$$^{**}$$	0.996	0.988	0.989
		(0.0115)	(0.0163)	(0.0205)	(0.0202)
Technology is a procedure	0.112$$^{***}$$				
	(0.0151)				
HTA in the year of inclusion	86.52$$^{***}$$	12.29$$^{***}$$	131.2$$^{***}$$	114.9$$^{***}$$	112.7$$^{***}$$
	(17.89)	(9.915)	(36.28)	(33.55)	(32.03)
WHO essential medicine				0.616$$^{*}$$	0.554$$^{**}$$
				(0.157)	(0.140)
Price cap regulation				1.301	1.202
				(0.313)	(0.290)
Only one firm per ATC5				0.878	0.797
				(0.191)	(0.187)
Years from registry oldest product				1.008	1.007
				(0.0166)	(0.0175)
Presence of generics				1.269	1.135
				(0.238)	(0.223)
Firms per ATC4 Last 2 years				1.004$$^{***}$$	
				(0.00153)	
Firms per ATC5 Last 2 years					1.010$$^{***}$$
					(0.00370)
Share institutional market ATC4 Last 2 years				6.323$$^{***}$$	
				(2.043)	
Share institutional market ATC5 Last 2 years					3.775$$^{***}$$
					(0.983)
IHH of both market ATC4 Last 2 years				1.000	
				(0.0000407)	
IHH of both market ATC5 Last 2 years					1.000
					(0.0000475)
Constant	0.0301$$^{***}$$	0.00183$$^{***}$$	0.0379$$^{***}$$	0.00394$$^{***}$$	0.00530$$^{***}$$
	(0.00434)	(0.000584)	(0.00573)	(0.00156)	(0.00230)
Observations	32024	21361	5519	5449	5449
Technologies included	7764	6268	1478	1455	1455
Pseudo-R2	0.296	0.0600	0.308	0.335	0.328

*Notes:* odds-ratios after a logistic regression which includes years fixed effects. Clustered at technology level (standard errors in parentheses). Significance: $$^{*}$$ $$p<0.1$$, $$^{**}$$ $$p<0.05$$, $$^{***}$$ $$p<0.01$$

The panel also shows that included technologies have a relatively high number of users in comparison with the average technology in the dataset. More expensive in general (but those included in 2017), and more likely to have undergone an HTA. We confirm this observation with the results of the logistic regression presented in Table 3. Column 1 refers to all technologies, column 2 only to procedures, and columns 3 to 5 only to medications. Columns 4 and 5 include pharmaceutical market characteristics in the regression, but as some medications might not be traded yearly, fewer observations are available.

Interestingly, for medications the number of users in the previous year reduced the odds of inclusion. Expenditure per user is associated with the odds of inclusion for procedures. The odds of inclusions are generally larger for medications than for procedures. What increases the chances of inclusion substantially is the presence of an HTA study at the time of the inclusion decision. In total, 224 technologies were assessed by the Colombian HTA agency in the study period, and 115 were eventually included (51.3%).

For the case of medications, the odds of inclusion are not linked to the concentration of the market, the time since registration in the country, or the presence or not of generics; if anything, it is higher when more firms are available. Three characteristics are relevant. First, products formerly regulated under the international reference pricing policy are more likely to be included in the HBP-CPU. Second, the share of the institutional sector (compulsory insurance companies, and health provider institutions) in the transactions: the larger it is, the higher the odds of inclusion. Third, essential medicines (according to the WHO model list) are less likely to be included. In 2012, 21% of these were not included in the HBP-CPU.

### Impacts of the inclusion

**Fig. 1:**
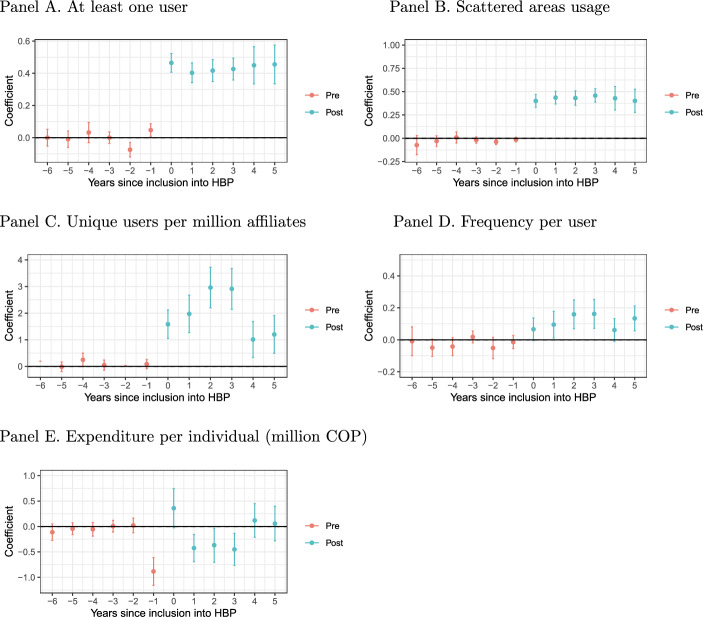
Impact of inclusion into HBP CS-DiD. Notes: coefficients obtained after a Callaway-Sant’Anna DiD. Dependent variables are transformed with the inverse hyperbolic sine transformation, therefore coefficients can be interpreted as percentage changes. Includes 95% confidence intervals after a bootstrap with 1,000 replications clustered at the technology level.

Figure 1 presents the results of the CS-DiD (detailed coefficients are in the Supplement Table G1). After the inclusion of one technology into the HBP-CPU, there is an increase of 43.6 pp. on the probability that the technology has at least one user per year, and a 42.6 pp. extra on the probability of such technologies being used in scattered areas of the country. For those technologies that have at least one user in the analysis period, there is an increase of 193.9% in users per million affiliates, 11.2% more frequency of use per technology. All these results were significant at the 95% confidence level.

Regarding expenditures per user, overall, there is no significant difference. Yet, in the first year, there is evidence of an increase of nearly 35% in the first year (not statistically significant), but then there is a decrease of nearly 40% for the next three periods. As observed in the Fig. 1, in the year before the inclusion there is a substantial reduction in the expenditures per individual (-88.5%). We cannot discard an anticipatory effect. The process of inclusion of health technologies for year *t* usually takes place in December of year $t-1$. However, the process takes several months as inclusions might be requested by pharmaceutical companies, scientific societies, or groups of patients; then the National Commission on Drug and Medical Devices Prices (which involves representatives of the ministries of finance, health, and trade) review the fiscal viability, and finally by the MHSP. In other words, it is not possible to fully anticipate the inclusion of a technology, but the actors might have a good guess that it could happen some months before the actual decision. Expenditures could be reduced by reduced frequencies per user (which we do not observe) or by reduced prices. Reduced prices could be the result of a bargaining process where insurance companies gain more power by the expectation of higher demand, or because providers want to press the actual inclusion by signaling that they can lower prices. We do not have evidence to disentangle the precise mechanism.

These results, in terms of usage (unique number of users, frequency peruse, and usage in scattered areas), align with the expectations. In terms of expenditures, we observe a reduction in costs per patient for some years but not for all, suggesting that the result depends on the specific inclusions. Considering results by cohort (Table G2 in the Supplement), we observe that increases in all usage variables are significant for inclusions in 2014, 2016, and 2019. The 2018 cohort also presents significant increases in the extensive margin but less clear in the intensive margin. Expenditure reductions come from the 2016 cohort while increases from the 2017 cohort. For 2019 there is a sharp reduction one year before the inclusions and an equivalent increase the year of the inclusion.

The results obtained with the synthetic control are similar (Supplement E). Although the magnitudes of the effects obtained in the two cases differ, the expected signs of the different effects estimated by both methods are congruent.

### Type of technology

**Fig. 2:**
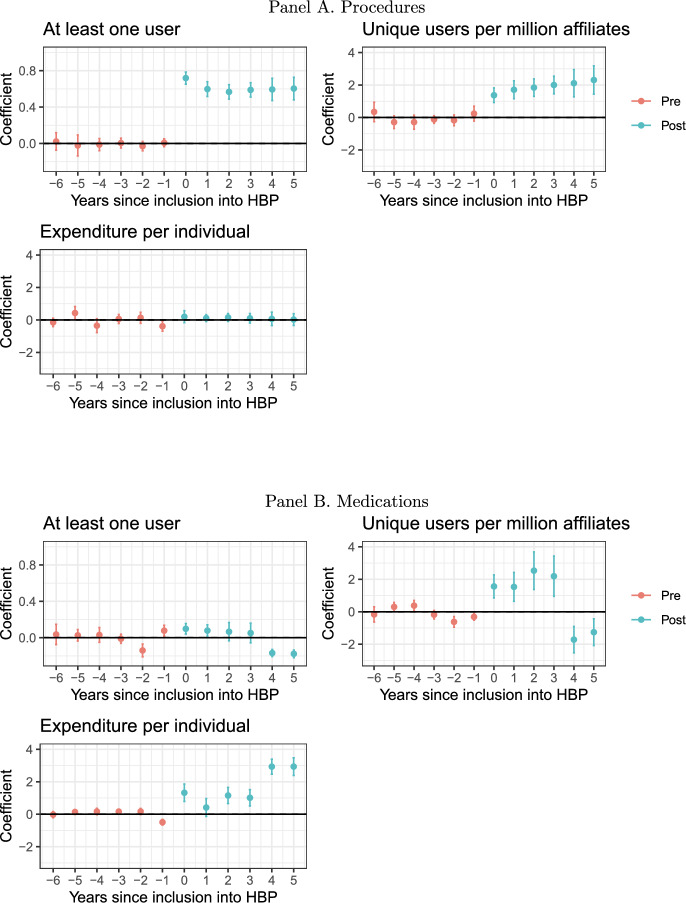
Dynamic effects CS-DiD by type of technology. Notes: coefficients obtained after a Callaway-Sant’Anna DiD. Includes 95% confidence intervals after a bootstrap with 1,000 replications clustered at the technology level

When we consider only procedures, there is a clear expansion on having at least one user (61.2 pp.) and on the number of users (188%), but no changes in terms of expenditures (see Figure 2 Panel A, or panel A of Supplement Table G3 for the coefficients). Results from the synthetic control are equivalent (see panel A of Supplement table E2 and panel A of Supplement figure E3). Still, we observe as well the one-year anticipatory effect (– 38.6%).

Instead, for the case of medications, there is no change in the probability of having at least one user but there is an increase in the unique users (80.58%). Rather than a decrease, we observe an increase in the expenditures per user of 162.7% (see Figure 2 Panel B, or Supplement Table G4 for the coefficients). Here we observe the anticipatory effect (– 49.2%).

For the case of medications it is also possible to consider if the IHH and share of the market that goes through the institutional market are modified. Overall, there is a positive increase in the IHH, mostly driven by the results in years four and five, and no changes on the channel through which users access the products.

As the DiD-CS analysis for medications indicated the presence of differential trends before the inclusion in some of the outcomes, using the synthetic control might be more adequate. Using this strategy, the increase in unique users is a bit larger (178% in the first year, and 143.6% in the second) but it is not significant at the 90% level. Yet, there is also an increase in expenditures per user of around 65% (see panel B of Supplement table E2 and Supplement figure E4). As with the DiD-CS, there are no changes in the institutional share but there is clear evidence of a reduction in the IHH.

### HTA status

**Fig. 3:**
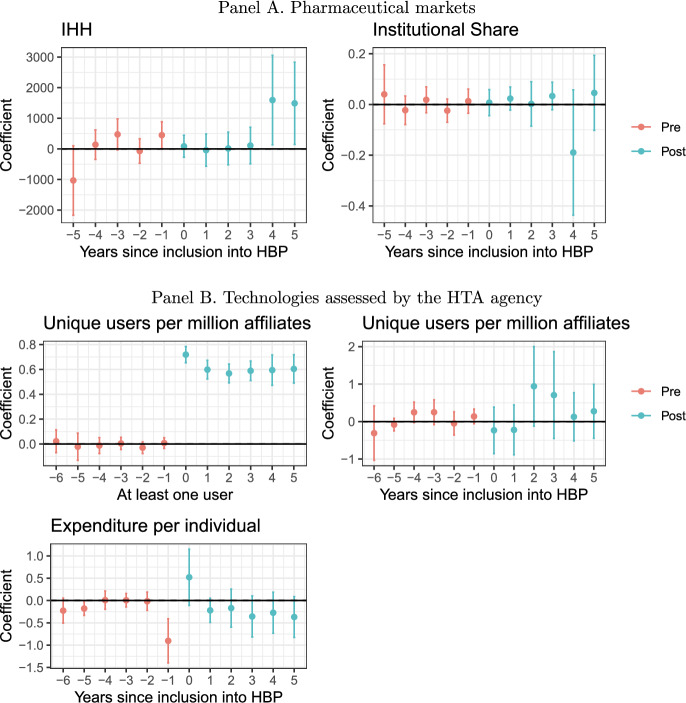
Dynamic effects CS-DiD on specific markets. Notes: markets are defined over ATC5. Coefficients obtained after a Callaway-Sant’Anna DiD. Includes 95% confidence intervals after a bootstrap with 1,000 replications clustered at the technology level.

Given the relevance of the HTA, Panel B of Figure 3 presents the main outcomes but considers only as treated those technologies that were included after the agency undertook an HTA process (panel B of Supplement Table G3). Inclusions in the period of analysis which the HTA agency did not review are not included in the sample. Overall, there is an average increase of 61 pp. in the probability that the technology has at least one user. However, for those with at least one unique user every year, there is no change in the number of unique users or expenditures. Yet, for expenditures, we observe a clear anticipatory effect, suggesting that insurance companies have a better guess of the inclusion, and the bargaining effect is amplified (nearly 90% reduction).

Results differ when using the synthetic control for those with at least one unique user every year (panel C of Supplement table E2 and panel B of Supplement figure E3). First, there is an increase in unique users of 20% in the first year and 45.4% in the second. Second, there is no change in usage in scattered areas. In addition, expenditures per user increased by around 105% in the first two years.

As a result, we conclude that there is an expansion in usage, but the impact on expenditures might be dependent of the actual comparison group.

## Discussion

This study analysed the effect of the periodic inclusion of new technologies in the HBP-CPU and its implications for the performance of the Colombian health system. Specifically, we examined changes in the frequency of use, access, geographical distribution, and expenditures per user. Our findings indicate that the inclusion of a health technology in the HBP-CPU leads to (i) an increase in the number of unique users accessing that technology, (ii) a broader geographical distribution, improving access in remote areas, and (iii) a higher frequency of use per patient. These results are expected and in line with the literature on insurance access and utilization of health services (Shami et al., 2019; Erlangga et al., 2019).

Regarding expenditures per user, results were mixed and appear to be technology-specific. While some cases showed a temporary increase in spending following inclusion, others exhibited cost reductions over time. This variation suggests that financial impacts depend on the nature of the technology, its market conditions, and existing price regulations. Notably, for procedures, no significant expenditure changes were observed, whereas, for medications, inclusion was associated with increased spending per user. This could be due to a combination of higher demand and limited competition in certain pharmaceutical markets.

This study has two key limitations related to data availability. First, although we measured access and expenditure, we could not evaluate whether the periodic updating of the HBP-CPU impacts health outcomes. Previous research highlights the importance of measuring long-term health benefits alongside financial considerations, yet we did not have access to adequate data to track these effects comprehensively. Second, we did not analyse the substitution patterns between newly included and existing technologies. This is particularly relevant in the pharmaceutical sector, where increased demand for recently introduced drugs may alter prescribing behaviours.

Given the study’s findings, several public policy recommendations can be made.

To enhance both health outcomes and financial sustainability, it is essential to prioritize cost-effective technologies while excluding those with limited therapeutic benefits. The updating process should be guided by robust HTA processes and economic evaluations, ensuring efficient resource allocation. While the presence of an HTA was a strong predictor of inclusion, very few included technologies had such evaluation prior to the inclusion. This highlights the need for systematic, evidence-based inclusion criteria.

Second, managing financial pressures requires specific policies. The inclusion of new health technologies did not lead to significant cost savings in the health system, in fact, there were higher expenditures per user despite an anticipatory reduction the year prior to the inclusion. Even in the case of medications-where distribution logistics are relatively straightforward-expenditures per user tended to increase. This suggests that price regulation and expenditure management mechanisms should be integrated into health coverage policies to control long-term financial pressures.

Third, an important part of the prioritisation system is the Colombian HTA Agency. A health technology assessment agency provides a country with legitimacy, scientific precision, and efficiency in the decision-making process on the set of health technologies to be financed. Our results show that there is an expansion in usage and inconclusive results on expenditures per user. Strengthening the HTA process could help optimize coverage decisions, ensuring that only technologies with proven cost-effectiveness and clinical value are publicly financed.

The periodic updating of HBP-CPU plays a crucial role in expanding access to essential health technologies, particularly in underserved regions. However, the impact on health expenditures remains complex, highlighting the need for complementary cost-containment strategies, such as dynamic pricing regulations and stronger prioritization frameworks. These findings contribute to the broader debate on how health benefit plans can be structured to balance access, equity, and financial sustainability in middle-income health systems.

## Supplementary Information

Below is the link to the electronic supplementary material.

Supplementary file 1 (pdf 2999 KB)

## Data Availability

The code for reproducing results is available at the following GitHub respository: https://github.com/androdri1/updatingColombianHBP. The data that support the findings of this study are available from Ministry of Health and Social Protection. Restrictions apply to the availability of these data, which were used under license for this study.

## Footnotes

1 The HBP-CPU represents more than 80% of the expenditure in the Colombian health system. That is slightly more than 11 billion US dollars (2019 prices) (Santa-María et al., 2021). Moreover, private voluntary health insurance covers less than 4% of the population (Casabianca et al., 2022).

2 The Colombia HBP has explicit exclusions, this list aims to avoid using public resources in technologies that are not directly related to improving the health of the patient or are considered inefficient as part of a particular treatment. Most of the technologies in the list are not the medication of procedure itself but are related to the specific purpose. For instance, the procedure “bilateral device augmentation mammoplasty” is excluded if it is for aesthetic purposes, but it is included for instance if its part of a breast cancer treatment. We do not study the impact of exclusions as we do not observe the purpose of usage. Hence, our strategy is not the most appropriate for measuring such effects. None of the technologies included are eventually excluded, so it does not introduce any bias in our estimates.

3 The standard strategy, the two-way fixed effects model, might deliver biased results under non-homogenous treatment (Athey & Imbens, 2021; Borusyak & Jaravel, 2017).
