# Supplementary material for: The impact of periodic updates to health benefits plan: access gains without cost savings?
Source: Int J Health Econ Manag. 2025 Apr 9;25(3):317–36. doi: 10.1007/s10754-025-09394-7 (PMC12568849; doi:10.1007/s10754-025-09394-7)
Supplement: Supplementary file 1 — (pdf 2999 KB) [file 10754_2025_9394_MOESM1_ESM.pdf]

# The impact of periodic updates to health benefits plan: Access gains without cost savings?

## Supplement

## A Criteria for updating PBS internationally and in Colombia

In this section, we contrast the Colombian HBP system with a group of countries from different continents: Canada, Chile, England, Ethiopia, India, Kenya, Malaysia, Mexico, Netherlands, Norway, and Uruguay. Lozano, Fullman, Mumford, Knight, and et al (2020) constructed an index (0 to 100) to rank the adequate coverage of a health system based on 23 indicators, including elements such as health promotion and treatment of diseases. Panel A of Table A1 shows that countries such as Ethiopia, India and Kenya have values of 52 or below, while others such as Canada, the Netherlands, or Norway have levels of 90 or above. Moreover, in all these countries except England, there is some explicit HBP in place.

In some countries, HBP plans can be decided at national or regional levels (Canada, Ethiopia, India). In other cases, only the administration occurs at the regional level (Mexico, Norway and New Zealand). For some countries, HBP applies only to specific population groups, like in Kenya; in others, it is the core of the entire health system, like in the Netherlands.

Regarding the number and nature of criteria on which HBP are defined and updated in the reference countries, these were divided into three distinct categories, following Hayati, Bastani, Kabir, Kavosi, and Sobhani (2018): intervention-related criteria, disease-related criteria, and community-related criteria. Panel B of Table A1 presents the fundamental criteria to define and update the HBP and our classification for each country.

In theory, almost all of these criteria are used in the definition and updating of HBP in virtually all countries included in the referencing. However, this may not be the case. In the literature consulted, it was impossible to establish the distance between official statements of intent and what happens in practice. On the other hand, some criteria often lack clear definitions or are difficult to instrumentalise and implement, such as sustainability, innovation, equity, affordability and access, insofar as they link debatable concepts, such as those associated with ethical and normative considerations in the value-related category with other concepts on which there is not complete agreement, such as health or sustainability.

The most widely used criteria were cost-effectiveness, effectiveness, budget impact and safety in the intervention-related category, equity, affordability and access in all countries included in the referencing. The disease burden is also widely used, especially in developing countries such as Chile, Mexico, Uruguay, Ethiopia, Kenya and India. On the other hand, innovation and disease severity are less used, mainly in high-income countries.

In summary, the overview of prioritization schemes in different countries suggests that although criteria and processes for developing HBP differ according to economic, social and cultural conditions and specific values of each country's society, there are standard criteria that inform and are used in the design, prioritization and updating of HBP: cost-effectiveness, clinical efficacy, the burden of disease, equity, financial protection and the effects on public and private budgets. Although these criteria are considered when updating the Colombian HBP, it is necessary to define a comprehensive and transparent methodological and institutional framework for updating, which does not currently exist. In the future, studies are also required on the determinants and factors of success or failure in the policies related to the HBP updating as a means to

achieve incremental improvements in the population's health through universal health coverage and different mechanisms for prioritizing and updating the HBP.

Table A1: Criteria for updating HBP in a selected group of countries

| | Canada | Chile | Colombia | Ethiopia | India | England | Kenya | Malaysia | Mexico | Norway | New Zealand | Netherlands | Uruguay |
|---|---|---|---|---|---|---|---|---|---|---|---|---|---|
| **Panel A. Characteristics of the system** | | | | | | | | | | | | | |
| Advance of the UHC | 90 | 74 | 74 | 47 | 47 | 88 | 52 | 67 | 61 | 94 | 83 | 90 | 69 |
| Explicit HBP | Yes | Yes | Mix | Yes | Yes | No | Yes | Yes | Yes | Yes | Yes | Yes | Yes |
| Central system | No | Yes | Yes | No | No | Yes | Yes | Yes | Mix | Mix | Mix | Yes | Yes |
| **Panel B. Determinant criteria of HBP** | | | | | | | | | | | | | |
| Intervention-related criteria | | | | | | | | | | | | | |
| Cost-effectiveness | X | X | X | X | X | X | X | X | X | X | X | X | X |
| Effectiveness | X | X | X | X | X | X | X | X | X | X | X | X | X |
| Budget impact | X | X | X | X | X | X | X | X | X | X | X | X | X |
| Safety | X | X | X | X | X | X | X | X | X | X | X | X | X |
| Sustainability | X | X | | X | X | X | X | X | X | X | X | | X |
| Cost of interventions | X | X | | X | X | X | X | X | X | X | X | X | X |
| Maximising the improvement of population health status | X | X | X | | | X | X | X | | X | X | X | X |
| Innovation | X | | | | | X | | X | | X | X | X | |
| Disease-related criteria | | | | | | | | | | | | | |
| Burden of disease | | X | X | X | X | | X | | X | | | | X |
| Severity of disease | | | X | X | X | | X | | X | | | | X |
| Community-related criteria | | | | | | | | | | | | | |
| Equity | X | X | X | X | X | X | X | X | X | X | X | X | X |
| Affordability | X | X | X | X | X | X | X | X | X | X | X | X | X |
| Access | X | X | X | X | X | X | X | X | X | X | X | X | X |

*Notes:* own elaboration based on Hayati et al. (2018) criteria categories. The advance of the UHC is measured with the effective coverage of health services index from Lozano et al. (2020).

# B The development and functioning of the Colombian HBP

From Law 100 of 1993, with the creation of the General System of Social Security in Health, guaranteed healthcare services were restricted to an explicit HBP-CPU, specific for CR (based on the *de facto* HBP of the former national health insurer) and a less broad one for the SR (based on prior social programs). The HBP-CPU defined a significant percentage of the national health budget, in addition to public health topics in charge of local governments. The Colombian regulatory framework also stated that the HBP-CPU must be updated. However, there was no indication of how to update each of them (Giedion, Panopoulou, & Gomez-Fraga, 2009; Giedion et al., 2014). The new health system based on structural pluralism began to increase health coverage in the general population. At the beginning of the 90s, only one in six people were part of the CR (Escobar, Giedion, Giuffrida, & Glassman, 2009). By 2008, nearly 90% of the population was already covered, and one out of three individuals was part of the RC, with one of the lowest out-of-pocket expenditure levels of the Americas (OECD, 2018).[1]

Over time, the lack of an integral and robust process to update the HBP-CPU resulted in two situations. First, insurers could pay for the provision of health technologies outside the HBP-CPU if a group of clinicians established a medical necessity. Another option was to ask for the technology to be provided via judiciary rulings (*tutelas*) to protect healthcare rights. In both cases, the government reimburses these resources outside their capitation contract. The flaws were not only to be found in the structure designed to provide the service but, on the one hand, in the absence of a legitimate, fairness, and efficient prioritisation scheme and, on the other, in the lack of technical capacity of the entities in charge of regulating and overseeing the system. This gave rise to grey areas, uncertainties, gaps, and accommodated interpretations in the HBP-CPU, resulting in the explosion of *recobros*, as a form of technical-administrative reimbursement mechanism (for the health insurers). These reimbursements were processed as *recobros*, essentially a fee-for-service mechanism. As a

[1] In Colombia, coinsurance is barely used, and copayments are established by the government as other price-related parameters of the managed care competition between health insurers (Buitrago, Amaya-Nieto, Miller, & Vera-Hernández, 2023).

result, the initial budget planning failed, and the system suffered problems related to financial sustainability, organisation, and functionality (Econometría, SEI, SIGIL, 2011).

In 2008 the Constitutional Court ruling T-760/2008 urged the government to solve the *recobros* crisis.[2] As a response, Law 1438 of 2011 established that the Ministry of Health and Social Protection (MHSP) had to undertake the responsibility of updating the HBP. Over the next years, the MHSP issued a series of normative resolutions to update the HBP-CPU.

Under the new scheme, updating the health technologies financed by the CPU in Colombia consists of 8 steps: i) identification of population needs in health; ii) selection of health technologies to be evaluated; iii) identification of safe and effective health technologies; iv) selection of health technologies to be included; v) presentation of the proposal for updating the commission and deliberations to formulate a recommendation for decision-making by the MHSP; vi) prior consultation of the proposal for the administrative act to the different actors of the General System of Social Security in Health, through the MHSP website; vii) administrative act signed by the Minister of Health and Social Protection and viii) the socialisation of the administrative act to different actors. Yet, steps (i) to (v) are not public, so the exact prioritisation criteria are not clear to the public and groups of patients or pharmaceutical companies would only know about the potential inclusion in the HBP-CPU once a draft of the administrative act was in place. For instance, it was not clear if the MHSP prioritized inclusions based on their declared criteria: cost-effectiveness, ii) effectiveness; iii) budget impact; iv) safety; v) burden of disease; vi) severity of disease; vii) equity; viii) affordability and ix) access (MinSalud, 2020). As part of the analysis, we established if particular observed characteristics of the technologies can predict actual inclusion into the HBP-CPU.

A major reform to the system reform took place in 2015 with the enactment of Law 1751 of 2015, a constitutional amendment that introduced a major change in the health system's setup. Instead of an explicit list of health benefits, the law stated that any technology could potentially be publicly funded unless it is explicitly defined that it shouldn't. However, the explicit list keeps being used to define the funding mechanism for technologies, the difference is that the *recobro* system was systematised using a digital tool managed by each prescribing physician (MIPRES). Only since 2020, the funding was assigned to a second capitation-based fund, directly controlled by each insurance company.

# C Construction of the dataset

| Variable name | Description | Source database |
|---|---|---|
| CODMUNI | Municipality code. Allows to identify the CPU analysis zones. | Suficiencia |
| FECHASERV | Date on which the health technology was required | Suficiencia |
| ACTIVIDAD | Identifies the health technology in demand | Suficiencia |
| VALORTOTAL | Expenditure on demanded health technology | Suficiencia |
| IDEANONIMA | User identifier. It allows to obtain the unique people who demanded health technologies. | Suficiencia |
| VALORRECOBRADO | Expenditure on demanded health technology | Recobros |
| MESSUMINISTRO | Month in which the health technology was required | Recobros |
| ANOSUMINISTRO | Year in which the health technology was required | Recobros |
| NITPROVEEDOR | Tax identification number of the service provider. It is used to identify the CPU analysis zones in this base. | Recobros |
| NOMBREPROVEEDOR | Name of service provider | Recobros |
| CODMEDSERPREST | Health technology code | Recobros |
| NOMMEDSERPREST | Health technology name | Recobros |

[2]The Court also requested the Government to match the HBP-CPU of both the CR and the SR. This process was finished by 2012, and it refers to the inclusion of technologies already in use in the HBP-CPU of the CR into the HBP-CPU of the SR. Therefore, we do not expect that process to affect our analyses. For a chosen set of 11 technologies, (Nuñez et al., 2015) found an increase in the use of health services by individuals in the SR.

Figure C1: Scattered areas

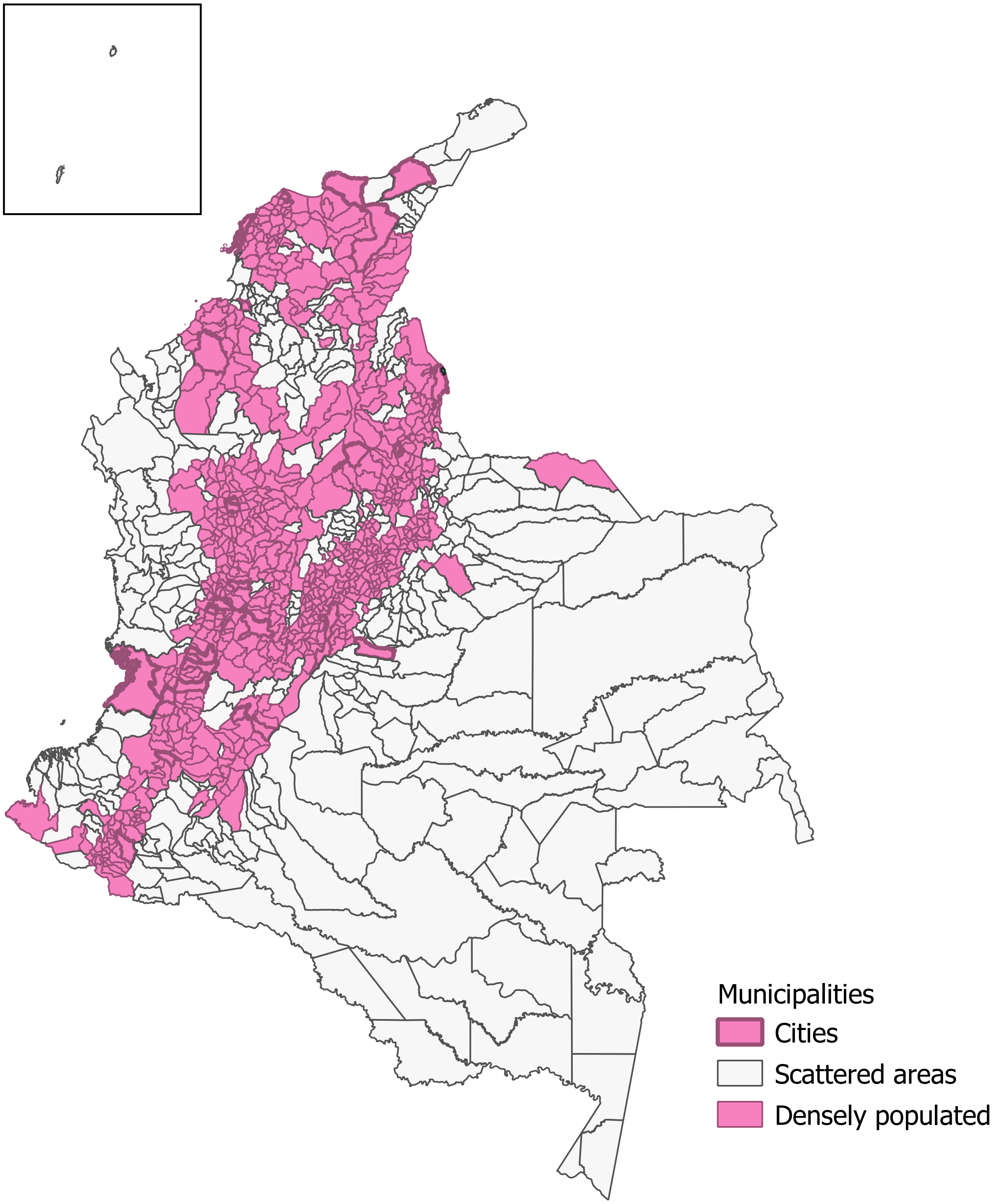

# D Case studies

A general review of the updates issued to the HBP-CPU analysed in this study showed that in 2014, the medicines financed with CPU resources were mainly immunosuppressive agents, immunostimulants, aromatase inhibitors and some antineoplastics, most of them used in the treatment of cancer and autoimmune diseases. Some active ingredients were also identified for treating epilepsy, depression and hormone therapy.

In 2016, the inclusion of medicines by reference subgroup (ATC 4) was mainly observed for insulins and analogues, non-selective beta-blocking agents, calcium channel blockers, proton pump inhibitors and statins. These medicines are used to manage chronic non-communicable diseases, such as diabetes, diseases of the cardiovascular system, gastrointestinal diseases and lipid disorders. Similarly, some fixed-dose combinations, such as nystatin plus metronidazole and alendronic acid plus vitamin D were identified.

Concerning 2017, the inclusions involve mainly solutions for peritoneal dialysis, some systemic antibiotics and other products such as powdered food with vitamins, iron and zinc were financed. An update was submitted in 2018 incorporating several medicines, including direct-acting antivirals, antiretrovirals for the treatment of human immunodeficiency virus (HIV), antineoplastic agents and immunomodulators for the treatment of cancer, of which four reference subgroups were funded, antithrombotic agents, antihypertensives, hormones, analgesics and medicines for the management of mental health pathologies (antidepressants, psychostimulants), the nervous system (selective serotonin agonists, anticholinergic and dopaminergic agents) and the alimentary tract and metabolism.

Finally, for 2019, the funding of many medicines is also highlighted. This update incorporated 15 reference subgroups, including antiemetics as adjuvant treatment in chemotherapy, hypoglycaemic drugs that exclude insulins for the management of diabetes and hypoglycaemia, aldosterone and angiotensin II antagonists for the treatment of hypertension, drugs for the management of benign prostatic hyperplasia, protease inhibitors for the treatment of HIV, and bisphosphonates for the prevention and treatment of bone resorption diseases. Urinary frequency and incontinence agents, beta-lactamase-sensitive penicillins and oxytocin analogues were also included in the subgroups. On the other hand, anxiolytics, anti-epileptics, antidepressants and drugs to improve gastrointestinal motility, among others, were also identified.

We present two medications and two procedures that were included in the HBP during the study period to illustrate the rationale of the inclusions. We will also explore the specific results for these four technologies.

i) The magnesium hydroxide (ATC5: A02AA04) was included in the HBP-CPU in 2016. A potential reason for its financing is its inclusion in the model list of essential medicines of the WHO within the gastrointestinal medicines used as antacids and other anti-ulcer drugs (WHO, 2007). However, other criteria considered for the prioritisation of technologies such as disease burden, epidemiological profile, local Clinical Practice Guideline (CPG) recommendations, costs, among others, cannot be ruled out (DROAS, 2020). Magnesium hydroxide as a monodrug in oral suspension is an effective pharmacological alternative to aluminium hydroxide, so its prescription and use as an antacid can be frequent in patients with gastrointestinal disorders, and its pharmaceutical form in suspension allows rapid action and easy administration in both adult and paediatric populations (Kluwer, 2021). This medication was not included in the price regulation during the study period and a HTA process was performed only one year after its inclusion. The product had been in the Colombian market for at least 20 years at the time of inclusion. Moreover, 29% of the transactions were in the institutional market, but in previous years the share was between 5% and 12%.

ii) Quetiapine (ATC5: N05AH04) is an atypical antipsychotic included in 2014, indicated in Colombia for the treatment of bipolar disorder, schizophrenia and major depressive disorder. It is also considered as an alternative treatment for generalised anxiety disorder when there has been an inadequate response to other medications. The use of quetiapine is recommended in lines of treatment in the local CPGs for depressive episode and recurrent depressive disorder and schizophrenia (MinSalud, Colciencias, IETS, 2014a, 2014b). It should be noted that other criteria such as disease status, protection of vulnerable groups, disease burden and epidemiological profile could be other considerations in favour of its funding and prescription in the health system (DROAS, 2020). Quetiapine has been registered in the market for 16 years at the year of inclusion in 2014. It was included in the price regulation scheme as well in 2014, even though the product had around 40 firms offering the product between 2012 and 2013, with a two-year window iHH of 2,619 (3,028 in a yearly basis). Around 44.7% of the sales were through the institutional section in that year. The product was considered as part of a HTA the same year of the inclusion.

iii) Stress echocardiography, either pharmacological or with exercise (CUPS 881210), is a procedure that involves administering a drug or subjecting a patient to physical activity to achieve myocardial stimulation and increase the heart rate to obtain ultrasound images of the heart to assess myocardial function (Kluwer, 2021; MinSalud, 2021). Its funding with CPU resources in 2014 and its increase in the number of services in

subsequent years could be attributed to the fact that its alternative, included since 2012, stress myocardial perfusion with pharmacological stress (CUPS 920408) or with rest and post-exercise (CUPS 920407), is a diagnostic procedure that requires special supplies and technical equipment to record the distribution of a radiopharmaceutical that allows assessment of the functioning and blood flow of the heart (Kluwer, 2021; MinSalud, 2021). This procedure is more difficult to access in its application because it requires specialised radiology services, whereas echocardiography may be a technique with better availability in health care services. Therefore, its coverage in the HBP led to an increase in its demand as it is considered a more available alternative to myocardial perfusion. On the other hand, it is possible financing with CPU resources could be based on access criteria, the situation of the health condition that requires the use of these procedures, and their costs, among other criteria (MinSalud, 2014).

iv) The revision and adjustment of external components of an implantable hearing device (CUPS 954903) is a procedure that consists of revising and adjusting the external components of the device according to the patient's needs (Kluwer, 2021; MinSalud, 2021). Its increased use may be because this procedure did not present any funded alternative within the HBP before 2016. Therefore, after its funding with CPU resources, it provided access to this service for people with implantable hearing devices and it was taken into account as a complementary procedure to be requested in the formulation of this type of hearing aid.

We can illustrate the results obtained in the main document for our four examples.

For medications, the Magnesium hydroxide was used in 2014(5) by around 11.77(18.06) individuals per million affiliates but this number grew up to 102.34 in 2016 (year of the inclusion) and even to 498.1 by 2019. Prior to 2016 the product was not used in in scattered areas of the country, but it has been the cases every year since. In terms of expenditures per capita, in 2014 it was of 8.87 USD per year and dropped to 3.84 USD since 2016. The Quetiapine was more common: it was used in scattered areas and had approximately 1,111 users per million affiliates in 2013, a number that drop to 458 in 2014, the year of the inclusion. However, by 2016 it had 2,638 users, and by 2019 it reached 5,374. In terms of expenditures, we observe a reduction from 60 USD in 2013 to 41 USD in 2014, and to 19 USD by 2019.

Concerning the procedures, the stress echocardiography, included in 2014, grew from 83.5 unique users per million affiliates in 2013 to 556.7 in 2014, 1,155.7 in 2015 and up to 2,073.2 in 2019. As well, it only became common its use in scattered areas since its inclusion in the HBP. As for expenditures per capita, it started on 155 USD in 2012, 73 USD in 2013, but returned to approximately 140 USD the rest of the years. The procedures around the implantable hearing device were very uncommon before 2016: they were used by about 0.05 individuals per million affiliates per year between 2012 and 2015. In 2016 the figure became 0.91, and by 2019 it was already 10.29. Expenditures per capita were highly variable, moving from 84 to 245 USD prior to 2016, and by 2016 the average was of 131 USD. However, in 2017 the figure jumped to 1,133 USD in 2017 and 1,424 in 2018, and only got back to 115 USD in 2019. This procedure became used in scatter areas only by 2019.

As for our examples, for the Magnesium hydroxide the market concentration was stable over time, as well as the proportion of it that was through the institutional market. However, for the Quetiapine, we observe a de-concentration of the market, moving from an IHH=3,028 in 2013 to 1,802 in 2014. Also the market went from being mostly 'private' (44,7%) to be dominated by the institutional sector (82.1%).

# E Synthetic control

## E.1 Method

The CS DiD presents striking differences in some of the outcomes, but there were doubts about the comparability of some technologies before the inclusion. For instance, the 2016 cohort includes technologies for which there is an important jump in frequencies and unique users (see Figure E1). For this reason, the synthetic control strategy will construct a comparison group where pre-trends are similar. In this analysis, we consider the sample of technologies that have at least one user every year in the study window.

Figure E1: Unique users and inclusion cohorts over time

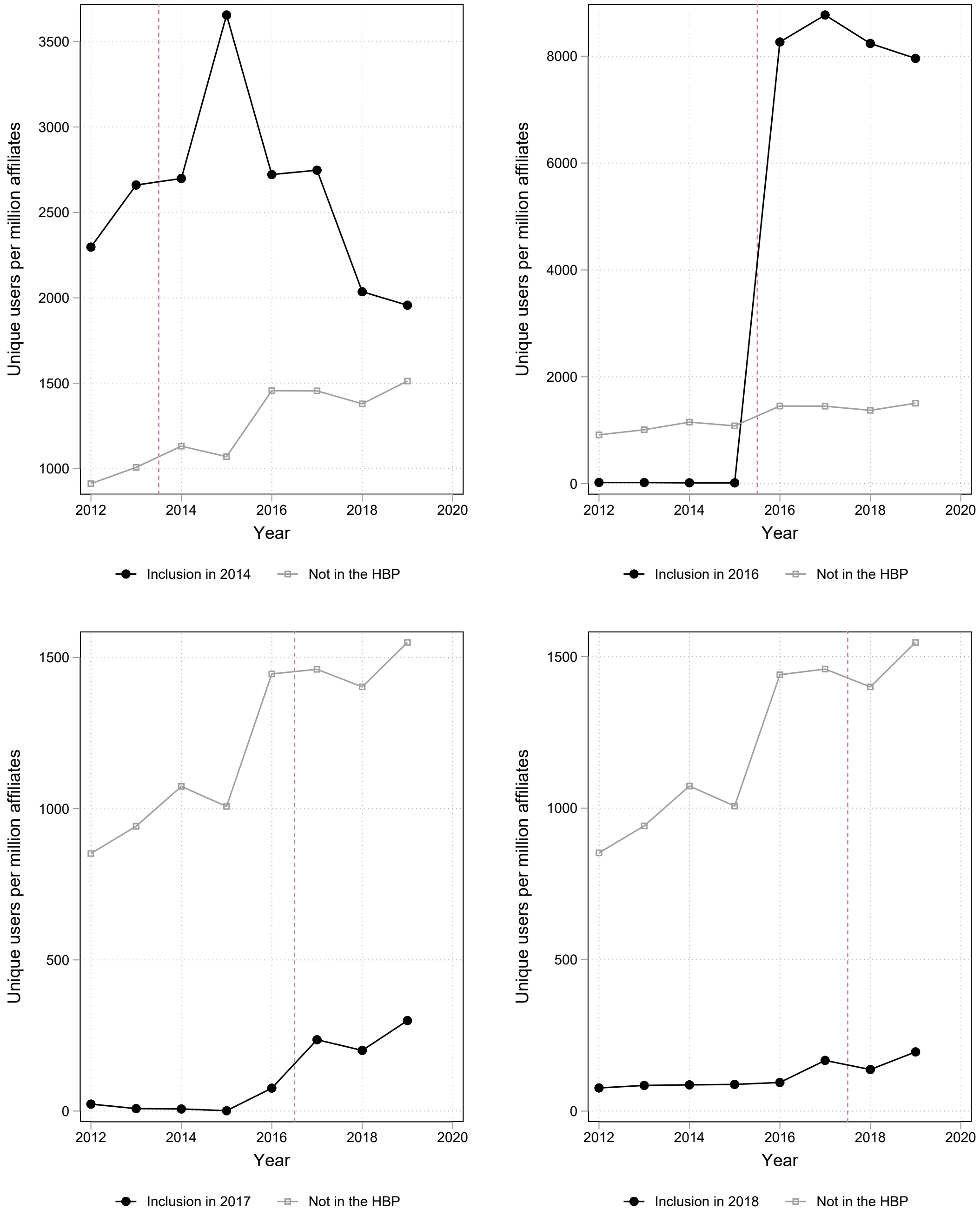


We follow Cavallo, Galiani, Noy, and Pantano (2010), who, in turn, extended Abadie and Gardeazabal (2003) and Abadie, Diamond, and Hainmueller (2010) work on synthetic controls for multiple treated units.

This strategy constructs a control group for each treated technology by weighting control technologies in such a way that they resemble the behaviour of the treated technology over time (before their introduction into the HBP-CPU) (Abadie, 2021).

If only one technology would be studied (unit i=1, one of the $G$ treated units), an estimate of the impact in year $\tau$ is obtained as follows:

$$\delta_{1,\tau} = Y_{1,\tau} - \frac{1}{G}\sum_{j=G+1}^{J} w_j^{(1)} Y_{j,\tau} \quad , \tag{1}$$

where weights $w_j^{(1)}$ are derived for each of the control technologies ($j \in \{G+1, J\}$). Weights are derived in this case from a constrained quadratic optimisation that minimises the distance $\sqrt{((X_1 - X_0W^{(1)})'V(X_1 - X_0W^{(1)})}$ where $W = \{w_j^{(1)}\}_{j=G+1}^{J}$ and $X_{k\in\{0,1\}}$ correspond to the variables to match. We match technologies on values of $Y$ prior to the inclusion into the HBP. Matrix $V$, a symmetric and positive semidefinite matrix, is chosen so that the root mean squared prediction error (RMSPE) of the pre-inclusion period is minimised.[3] The procedure outlined above for a single treated unit can be extrapolated to multiple treatment units, as in the case of Cavallo et al. (Cavallo et al., 2010). As a result, a general estimate can be obtained:

$$\delta_{\tau} = \frac{1}{G}\sum_{j=1}^{G} Y_{j,\tau} - \frac{1}{G}\sum_{j=1}^{G}\sum_{k=G+1}^{J} w_k^{(j)} Y_{k,\tau}. \tag{2}$$

Given a large number of potential alternatives for the synthetic control, we considered for the algorithm only the three closest neighbours for each included technology in terms of their pre-trends for the four outcomes in two years prior to inclusion (both in levels and their transformations). The selection was based on the propensity score (Leuven & Sianesi, 2003).

For inference, the standard strategy is to use a permutation test. It is implemented by repeating the analysis described above for each of the control units as if they were treated (as placebos) to compute an empirical distribution of the random differences that result from the procedure. This distribution allows us to test if the estimated effects are statistically different from the placebo results.

## E.2 Main results

Table E1 presents the results per cohort for the proposed outcome variables, which are presented in levels. Figures E5 and E6 show the average time trends of the included technologies and their synthetic controls.

First, it presents those included by the resolution of 2013 and which entered effectively in 2014 into the HBP-CPU. There is evidence of an increase in the total number of users per affiliate all periods after the inclusion In terms of frequency, the increase is significant for the second to the fourth years. In the case of expenditures, the increase is significant in three years, but the non-significant years present similar coefficients. Estimates for usage in scattered areas for the first two years are significant, the other four have a larger coefficient but the estimates are not significant. It is important to make it clear that the results in scattered areas here are different from the analysis with the CS main exercise as for the synthetic control we are using a restricted sample (technologies with users all years) which is not the case for the CS estimates.

Second, the 2016 inclusions resulted in a substantial increase in users and usage in scattered areas, but not in frequencies. For expenditures, there is evidence of an increase two and three years after the inclusion.

Third, for the 2017 inclusions, there are no significant effects in terms of unique users, frequency per user, or expenditures per user. Yet, there is a large increase in the probability of use in scattered areas.

Fourth, for the 2019 inclusions, there is an increase in all variables of interest.

Lastly, the table presents the overall results (see Figure E2 as well), which consider all but the 2019 cohort (so we can have at least two periods). While year-specific estimates are not significant (only frequency per user in the second year), the joint permutation test of the impact shows that for all outcomes there is a significant difference between treatment and control after the inclusions but not before the inclusions. We found that for the first two years, there was an increase of nearly 200% in unique users, around 7% in frequency per user, 22% in costs per user, and 38 pp. in usage in scattered areas.

[3] The RMSPE is computed with the same distance described before, but $X$ corresponds to the value of the outcome variable at $t = \tau$.

## E.3 Results by type of technology and HTA status

Considering only procedures (see Panel A of Table E2 and Panel A of Figure E3), the increase in unique users is large (around +143%) but is not significant (although the p-values are 0.15). For frequency, there is an increase of 5.9% in the second year. For scattered areas usage, there is an increase of +30 pp. For expenditures, no coefficient is significant.

As for medications (see Panel B of Table E2 and Figure E4), there is an increase in expenditures (+67%) and usage in scattered areas (+40 pp.). For the unique users and frequencies, increases are not significant but as well p-values are close to 0.10. The extra result is a clear reduction in the industrial concentration of 236 units (the measure goes from 0 to 10.000 -monopoly-) which is not particularly large.

Finally, when considering only technologies with HTA (see Panel C of Table E2 and Panel B of Figure E3), results are in line with the main results but estimates are smaller in magnitude.

Table E1: Impact of inclusion into HBP synthetic control: group-time average treatment effects

| | | Unique users per million affiliates | | | Frequency per user | | | Expenditure per user (million COP) | | | Scattered areas (1: Yes, 0 No) | | |
|---|---|---|---|---|---|---|---|---|---|---|---|---|---|
| Group | Year/Lead | ATT | p-value | | ATT | p-value | | ATT | p-value | | ATT | p-value | |
| 2014 | 2014 | 2.929 | < 0.001 | * | 0.067 | 0.005 | * | 1.063 | 0.725 | | 0.171 | < 0.001 | * |
| 2014 | 2015 | 3.291 | < 0.001 | * | -0.035 | 0.534 | | 1.183 | 0.008 | * | 0.342 | < 0.001 | * |
| 2014 | 2016 | 2.834 | < 0.001 | * | 0.155 | 0.277 | | 1.541 | 0.088 | | 0.6 | 0.57 | |
| 2014 | 2017 | 2.344 | 0.001 | * | 0.253 | 0.454 | | 1.772 | 0.548 | | 0.691 | 0.269 | |
| 2014 | 2018 | 2.642 | < 0.001 | * | 0.175 | 0.224 | | 2.833 | < 0.001 | * | 0.638 | 0.583 | |
| 2014 | 2019 | 3.373 | < 0.001 | * | 0.244 | 0.006 | * | 2.323 | < 0.001 | * | 0.625 | 0.441 | |
| 2016 | 2016 | 3.895 | < 0.001 | * | 0.13 | 0.946 | | 0.334 | 0.999 | | 0.476 | 0.011 | * |
| 2016 | 2017 | 3.115 | < 0.001 | * | 0.126 | 0.997 | | 0.054 | 1 | | 0.565 | 0.011 | * |
| 2016 | 2018 | 2.99 | < 0.001 | * | 0.018 | 0.666 | | 1.057 | < 0.001 | * | 0.491 | 0.037 | * |
| 2016 | 2019 | 3.369 | < 0.001 | * | 0.087 | 0.959 | | 1.39 | < 0.001 | * | 0.506 | 0.011 | * |
| 2017 | 2017 | 7.355 | 0.078 | | -0.007 | 0.873 | | 0.599 | 0.584 | | 0.981 | 0.048 | * |
| 2017 | 2018 | 7.012 | 0.113 | | 0.021 | 0.645 | | 0.413 | 0.67 | | 0.972 | 0.042 | * |
| 2017 | 2019 | 7.465 | 0.149 | | 0.056 | 0.442 | | 0.69 | 0.53 | | 0.957 | 0.129 | |
| 2018 | 2018 | 0.645 | 0.947 | | 0.196 | 0.126 | | 4.133 | < 0.001 | * | 0.725 | < 0.001 | * |
| 2018 | 2019 | 1.255 | 0.371 | | 0.405 | < 0.001 | * | 4.65 | < 0.001 | * | 0.736 | < 0.001 | * |
| 2019 | 2019 | 4.138 | < 0.001 | * | 0.19 | < 0.001 | * | 3.781 | < 0.001 | * | 0.781 | < 0.001 | * |
| Overall | 0 (1$^{st}$) | 2.1 | 0.524 | | 0.069 | 0.23 | | 0.116 | 0.172 | | 0.384 | 0.177 | |
| Overall | 1 (2$^{nd}$) | 1.923 | 0.478 | | 0.082 | 0.013 | * | 0.328 | 0.159 | | 0.396 | 0.291 | |
| p-val all post | | < 0.001 | | | < 0.001 | | | < 0.001 | | | < 0.001 | | |
| p-val all pre | | 0.99999 | | | 0.703596 | | | 1 | | | 0.117 | | |

**Notes:** coefficients obtained after a synthetic control implemented with *synth_runner* package in Stata 18. Robust p-values are derived from permutation tests after 1'000,000 placebo averages. Significant at 95% level: *. For the overall results, we present the joint p-value of the presence of a difference after the inclusion (p-val. all post) and the joint p-value of a difference before the inclusion (p-val all pre).

Table E2: Impact of inclusion into HBP synthetic control: additional exercises

**A. Only health procedures**

| | Unique users per million affiliates | | | Frequency per user | | | Expenditure per user (million COP) | | | Scattered areas (1: Yes, 0 No) | | |
|---|---|---|---|---|---|---|---|---|---|---|---|---|
| Lead | ATT | p-value | | ATT | p-value | | ATT | p-value | | ATT | p-value | |
| 0 ($1^{st}$) | 1.37 | 0.151 | | 0.073 | 0.28 | | -0.036 | 0.111 | | 0.287 | < 0.001 | * |
| 1 ($2nd$) | 1.487 | 0.151 | | 0.059 | < 0.001 | * | 0.117 | 0.308 | | 0.339 | 0.012 | * |

**B. Only medications**

| | Unique users per million affiliates | | | Frequency per user | | | Expenditure per user (million COP) | | | Scattered areas (1: Yes, 0 No) | | |
|---|---|---|---|---|---|---|---|---|---|---|---|---|
| Lead | ATT | p-value | | ATT | p-value | | ATT | p-value | | ATT | p-value | |
| 0 ($1^{st}$) | 1.782 | 0.138 | | 0.008 | 0.116 | | 0.677 | 0.001 | * | 0.416 | 0.001 | * |
| 1 ($2^{nd}$) | 1.436 | 0.12 | | 0.061 | 0.109 | | 0.622 | < 0.001 | * | 0.398 | 0.01 | * |

| | IHH (Percent P) | | | Institutional share (0 - 1) | |
|---|---|---|---|---|---|
| Lead | ATT | p-value | | ATT | p-value |
| 0 ($1^{st}$) | -236.369 | < 0.001 | * | 0.039 | 0.332 |
| 1 ($2^{nd}$) | -226.298 | < 0.001 | * | 0.05 | 0.339 |

**C. Only for technologies with HTA**

| | Unique users per million affiliates | | | Frequency per user | | | Expenditure per user (million COP) | | | Scattered areas (1: Yes, 0 No) | | |
|---|---|---|---|---|---|---|---|---|---|---|---|---|
| Lead | ATT | p-value | | ATT | p-value | | ATT | p-value | | ATT | p-value | |
| 0 ($1^{st}$) | 0.203 | 0.086 | | 0.074 | 0.13 | | 1.054 | 0.056 | | 0.32 | 0.504 | |
| 1 ($2^{nd}$) | 0.455 | 0.019 | * | 0.163 | 0.365 | | 1.11 | < 0.001 | * | 0.276 | 0.599 | |

**Notes:** coefficients obtained after a synthetic control implemented with *synth_runner* package in Stata 16. Robust p-values are derived from permutation tests after 1'000,000 placebo averages. Significant at 95% level: *.

Figure E2: Outcomes over time: inclusions and their synthetic control

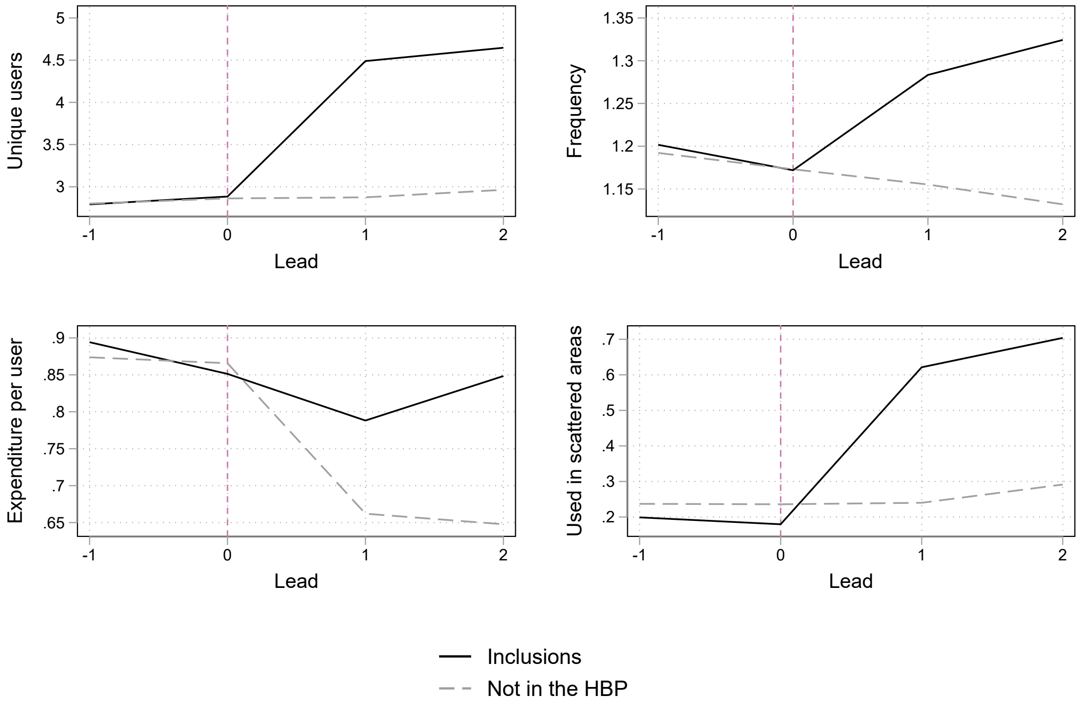

Figure E3: Synthetic control: impact only for procedures and for technologies with an HTA

Panel A. Health procedures only

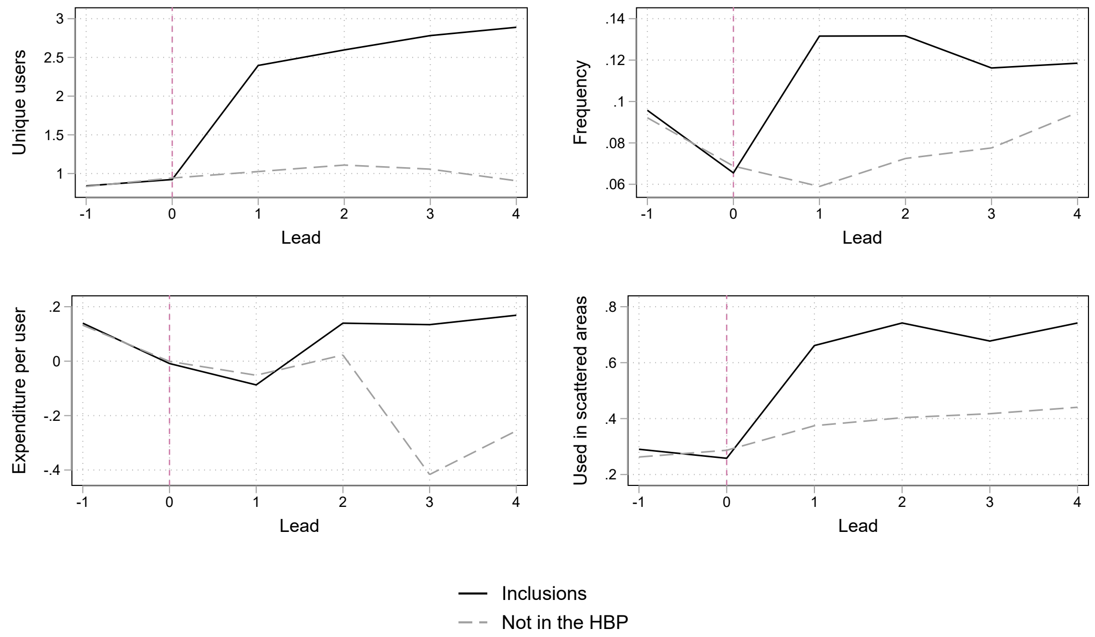


Panel B. Only technologies with HTA prior to inclusion

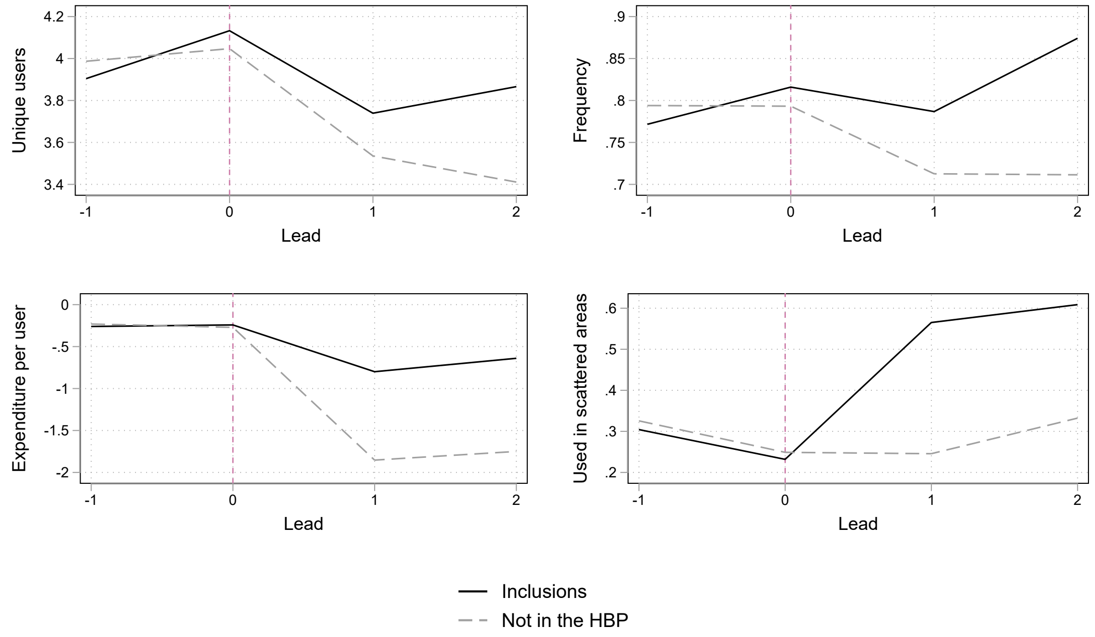

Figure E4: Synthetic control: impact only for medications

Panel A. Main outcomes

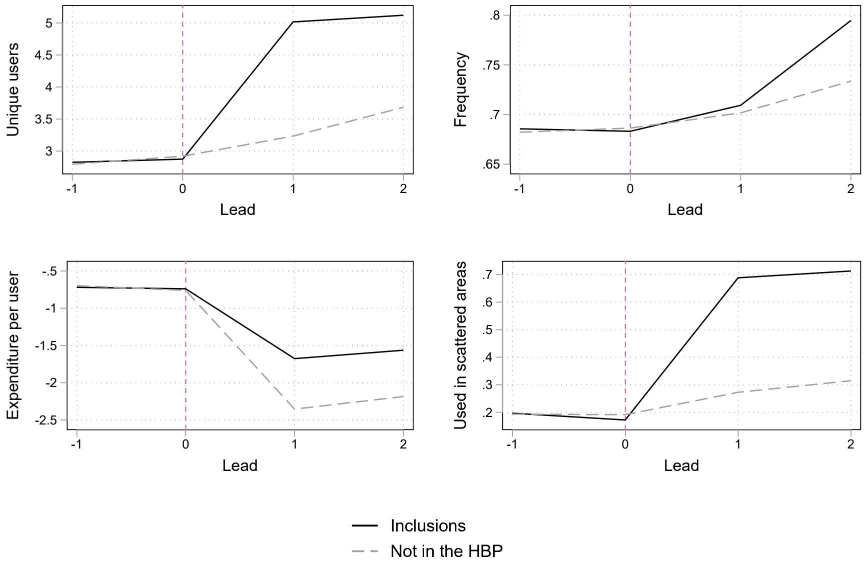


Panel B. Pharmaceutical market outcomes

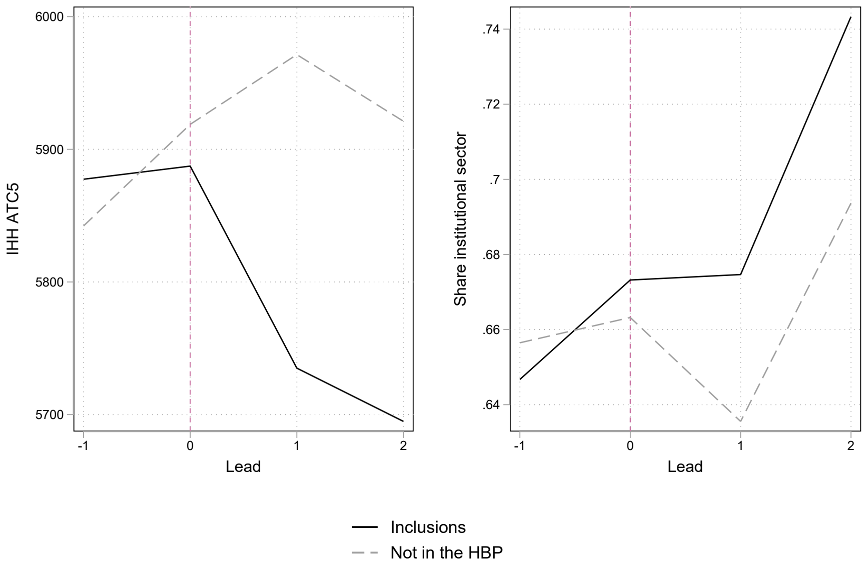

Figure E5: Synthetic control: group-time trends (I)
Panel A. Inclusions in 2014

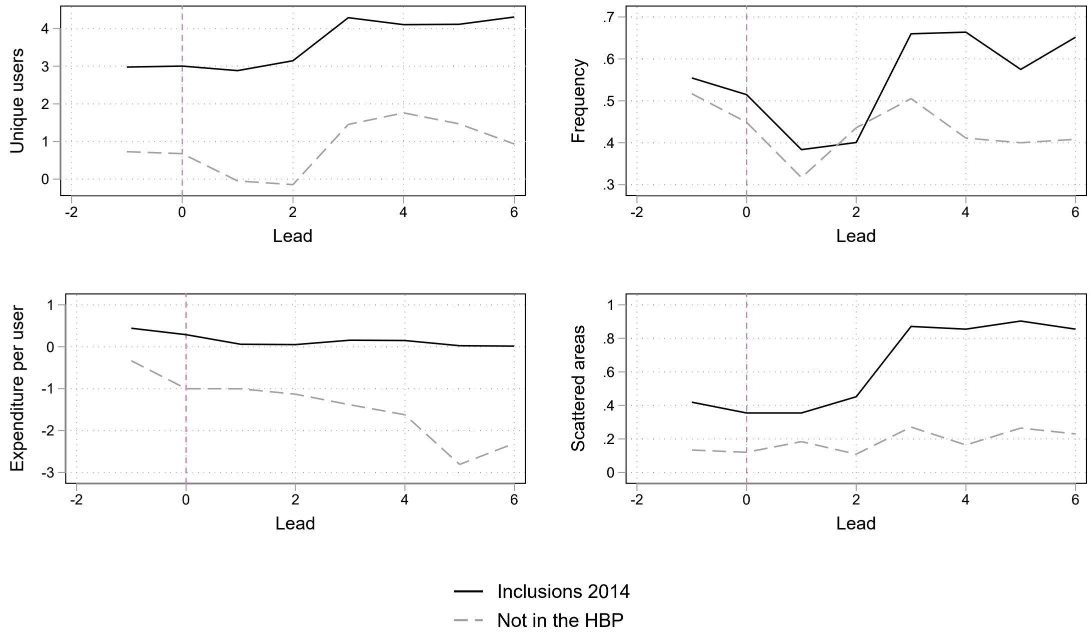


Panel B. Inclusions in 2016

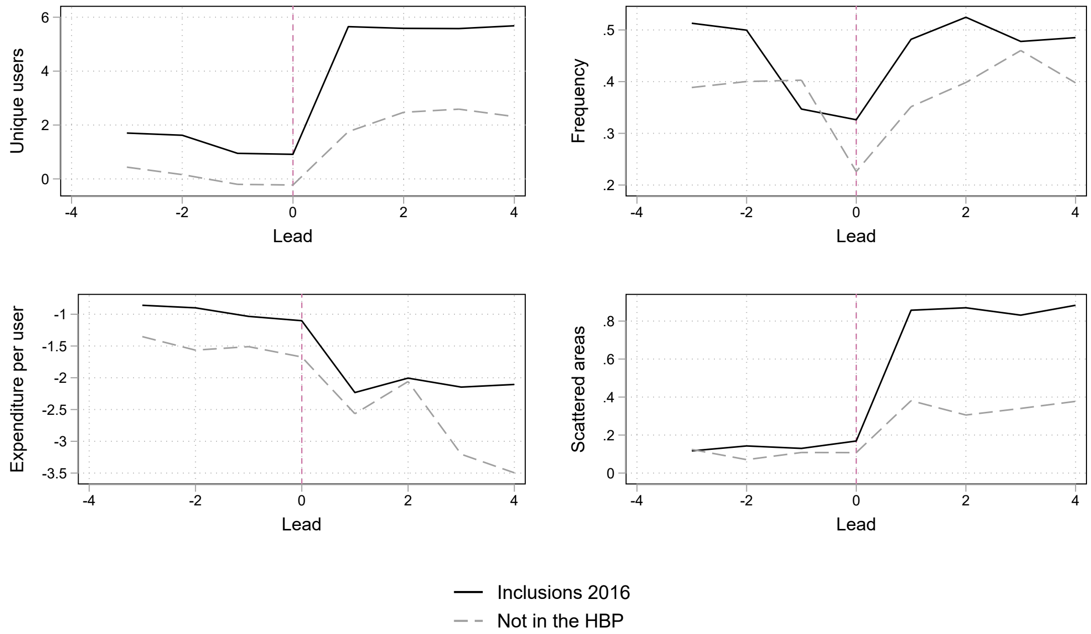

Figure E6: Synthetic control: group-time trends (II)

Panel A. Inclusions in 2017

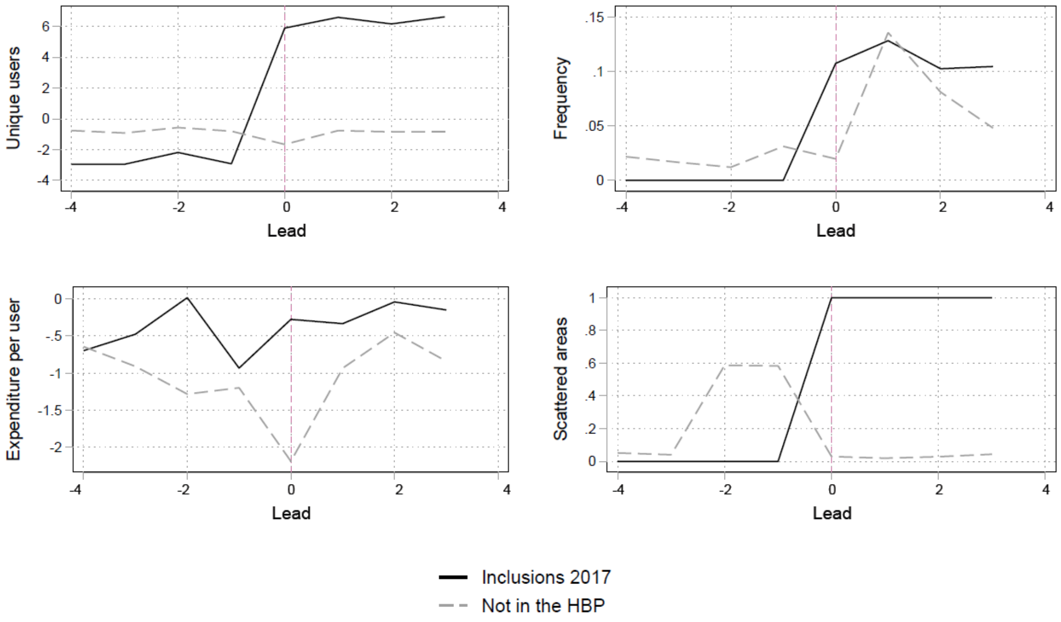


Panel B. Inclusions in 2018

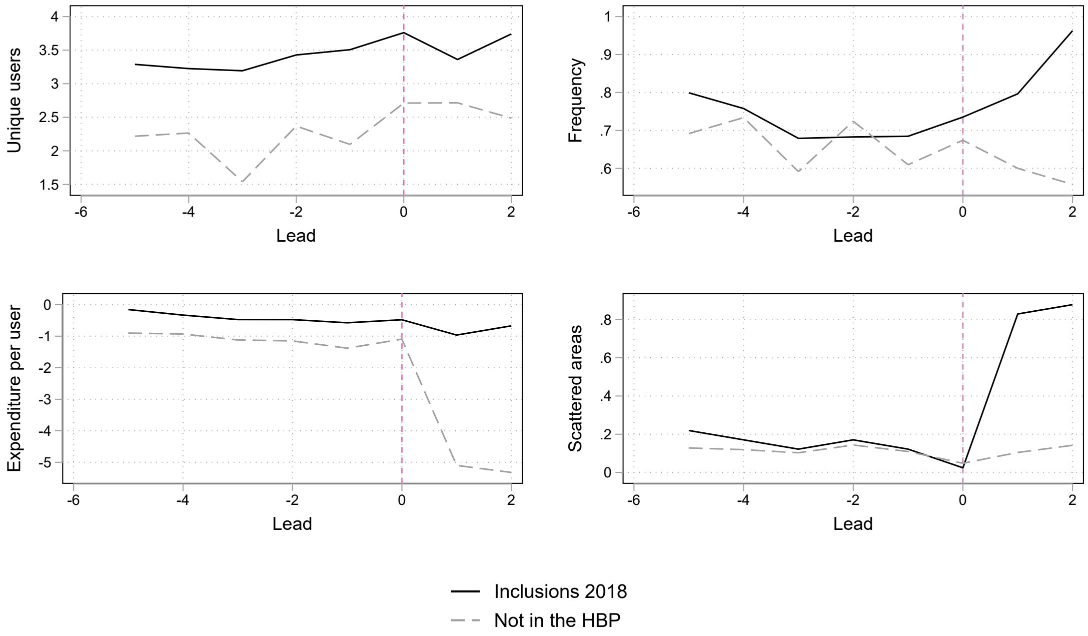

# F Additional tables and figures

Table F1: Impact of inclusion into HBP Callaway-Sant'Anna DiD

| | At least one user (1: Yes, 0 No) | | | Scattered areas (1: Yes, 0 No) | | | Unique users per million affiliates | | | Frequency per user | | | Expenditure per user (million COP) | | |
|---|---|---|---|---|---|---|---|---|---|---|---|---|---|---|---|
| time | ATT | Std. Error | | ATT | Std. Error | | ATT | Std. Error | | ATT | Std. Error | | ATT | Std. Error | |
| -6 | 0.0001 | 0.0197 | | -0.0736 | 0.0366 | | -0.2333 | 0.159 | | -0.0094 | 0.0368 | | -0.1132 | 0.0645 | |
| -5 | -0.0091 | 0.0178 | | -0.0299 | 0.019 | | -0.0137 | 0.0672 | | -0.0496 | 0.0207 | | -0.044 | 0.0472 | |
| -4 | 0.0321 | 0.0215 | | 0.0087 | 0.021 | | 0.2478 | 0.0943 | | -0.0423 | 0.021 | | -0.0556 | 0.0571 | |
| -3 | 0.0002 | 0.0136 | | -0.0191 | 0.0111 | | 0.0496 | 0.0602 | | 0.0178 | 0.015 | | 0.0056 | 0.0415 | |
| -2 | -0.0748 | 0.0167 | * | -0.0379 | 0.011 | * | -0.2193 | 0.0909 | | -0.0513 | 0.0275 | | 0.0204 | 0.0605 | |
| -1 | 0.0469 | 0.0132 | * | -0.0148 | 0.0095 | | 0.0877 | 0.0649 | | -0.0147 | 0.0163 | | -0.8848 | 0.1112 | * |
| 0 ($1^{st}$) | 0.4646 | 0.0199 | * | 0.4009 | 0.0226 | * | 1.5839 | 0.2002 | * | 0.0658 | 0.0284 | | 0.3592 | 0.1398 | |
| 1 ($2^{nd}$) | 0.403 | 0.0228 | * | 0.4357 | 0.0242 | * | 1.9723 | 0.2451 | * | 0.0947 | 0.0347 | * | -0.4249 | 0.1007 | * |
| 2 ($3^{rd}$) | 0.4167 | 0.0256 | * | 0.4315 | 0.026 | * | 2.9604 | 0.2982 | * | 0.1593 | 0.0346 | * | -0.3682 | 0.1227 | * |
| 3 ($4^{th}$) | 0.4263 | 0.0253 | * | 0.4581 | 0.0257 | * | 2.9097 | 0.2871 | * | 0.162 | 0.0354 | * | -0.4508 | 0.1313 | * |
| 4 ($5^{th}$) | 0.4495 | 0.0434 | * | 0.4289 | 0.0425 | * | 1.0089 | 0.2342 | * | 0.0606 | 0.0304 | | 0.1189 | 0.1252 | |
| 5 ($6^{th}$) | 0.4554 | 0.0431 | * | 0.4015 | 0.0427 | * | 1.1994 | 0.2479 | * | 0.1339 | 0.0317 | * | 0.0571 | 0.1298 | * |
| Overall | 0.4359 | 0.0245 | * | 0.4261 | 0.0214 | * | 1.9391 | 0.1984 | * | 0.1127 | 0.0222 | * | -0.1181 | 0.0876 | * |

**Notes:** coefficients obtained after a Callaway-Sant'Anna DiD. The variables of the Unique users per million affiliates, frequency per user, and expenditure per user are for technologies that had at least one user all years and are expressed in logarithms, therefore, coefficients can be interpreted as percentage changes. Standard errors in parentheses are computed with a bootstrap with 1,000 replications clustered at the technology level. Significant at 95% level: *.

Table F2: Impact of inclusion into HBP Callaway-Sant'Anna DiD: group-time average treatment effects

| | | At least one user (1: Yes, 0:No) | | | Scattered areas (1: Yes, 0:No) | | | Unique users per million affiliates | | | Frequency per user | | | Expenditure per user (million COP) | | |
|---|---|---|---|---|---|---|---|---|---|---|---|---|---|---|---|---|
| Group | Time | ATT | Std. Error | | ATT | Std. Error | | ATT | Std. Error | | ATT | Std. Error | | | | |
| 2014 | 2013 | 0.0173 | 0.0267 | | -0.0375 | 0.0247 | | -0.0578 | 0.0642 | | -0.0408 | 0.0179 | | -0.131 | 0.0607 | |
| 2014 | 2014 | 0.5502 | 0.0408 | * | 0.0836 | 0.0441 | | -0.0381 | 0.2364 | | -0.1147 | 0.0372 | * | -0.091 | 0.0897 | |
| 2014 | 2015 | 0.507 | 0.0411 | * | 0.245 | 0.0493 | * | 0.2651 | 0.2944 | | -0.1389 | 0.0396 | * | -0.2084 | 0.0977 | |
| 2014 | 2016 | 0.4437 | 0.0388 | * | 0.3762 | 0.04 | * | 1.1327 | 0.2048 | * | 0.143 | 0.0334 | * | 0.0019 | 0.1157 | |
| 2014 | 2017 | 0.4324 | 0.0406 | * | 0.3583 | 0.0453 | * | 0.9366 | 0.2322 | * | 0.1382 | 0.0304 | * | -0.1446 | 0.1285 | |
| 2014 | 2018 | 0.4495 | 0.041 | * | 0.4289 | 0.039 | * | 1.0089 | 0.2378 | * | 0.0606 | 0.0279 | | 0.1189 | 0.1367 | |
| 2014 | 2019 | 0.4554 | 0.0405 | * | 0.4015 | 0.0413 | * | 1.1994 | 0.254 | * | 0.1339 | 0.0325 | * | 0.0571 | 0.1337 | |
| 2016 | 2013 | -0.007 | 0.0192 | | -0.0069 | 0.0126 | | -0.1742 | 0.0868 | | -0.0141 | 0.0253 | | -0.0185 | 0.0813 | |
| 2016 | 2014 | -0.1173 | 0.0252 | * | -0.0078 | 0.0102 | | -0.5893 | 0.1602 | * | -0.1342 | 0.0413 | * | 0.0065 | 0.1034 | |
| 2016 | 2015 | 0.0824 | 0.0209 | * | -0.0105 | 0.0088 | | 0.0255 | 0.0976 | | -0.0615 | 0.036 | | -0.1854 | 0.0857 | |
| 2016 | 2016 | 0.5136 | 0.0302 | * | 0.445 | 0.0307 | * | 4.394 | 0.3863 | * | 0.1786 | 0.061 | * | -1.0098 | 0.205 | * |
| 2016 | 2017 | 0.4245 | 0.0302 | * | 0.4826 | 0.0311 | * | 4.31 | 0.3861 | * | 0.2095 | 0.0603 | * | -0.9241 | 0.2028 | * |
| 2016 | 2018 | 0.4091 | 0.0336 | * | 0.4604 | 0.03 | * | 4.3793 | 0.3806 | * | 0.1746 | 0.0604 | * | -0.6809 | 0.217 | * |
| 2016 | 2019 | 0.4232 | 0.0309 | * | 0.5101 | 0.0306 | * | 4.4781 | 0.3754 | * | 0.1808 | 0.0561 | * | -0.6941 | 0.2102 | * |
| 2017 | 2013 | -0.1323 | 0.182 | | -0.0105 | 0.0039 | | -0.5433 | 0.3381 | | -0.2603 | 0.378 | | 0.0627 | 0.1377 | |
| 2017 | 2014 | 0.0165 | 0.0032 | * | -0.0042 | 0.0038 | | 0.0112 | 0.6208 | | 0.1076 | 0.1272 | | 0.1496 | 0.3582 | |
| 2017 | 2015 | 0.1266 | 0.1816 | | -0.021 | 0.0039 | * | -0.0439 | 0.4826 | | -0.0565 | 0.0145 | * | -0.3486 | 0.5287 | |
| 2017 | 2016 | 0.0931 | 0.182 | | 0.0984 | 0.1804 | | 3.6839 | 3.5967 | | 0.0003 | 0.1817 | | 0.2232 | 0.4049 | |
| 2017 | 2017 | 0.2811 | 0.1846 | | 0.4309 | 0.1732 | | 4.0697 | 2.512 | | 0.0853 | 0.101 | | -0.1081 | 0.0692 | |
| 2017 | 2018 | 0.1757 | 0.1815 | | 0.4475 | 0.1747 | | 3.9169 | 2.6648 | | 0.0268 | 0.0339 | | 0.3653 | 0.0901 | * |
| 2017 | 2019 | 0.1545 | 0.2447 | | 0.4268 | 0.1758 | | 4.277 | 2.5932 | | 0.0671 | 0.096 | | 0.352 | 0.0299 | * |
| 2018 | 2013 | -0.0144 | 0.0359 | | -0.023 | 0.0234 | | -0.1289 | 0.0713 | | -0.0414 | 0.0285 | | -0.151 | 0.0731 | |
| 2018 | 2014 | 0.029 | 0.0481 | | -0.0417 | 0.0318 | | 0.1114 | 0.0964 | | -0.0524 | 0.0255 | | 0.0504 | 0.1016 | |
| 2018 | 2015 | 0.0588 | 0.0325 | | 0.004 | 0.0313 | | 0.3326 | 0.1606 | | -0.024 | 0.0247 | | -0.1022 | 0.0697 | |
| 2018 | 2016 | 0.0003 | 0.0288 | | -0.0694 | 0.0308 | | -0.1836 | 0.1122 | | 0.0273 | 0.0436 | | 0.0045 | 0.0811 | |
| 2018 | 2017 | 0.0079 | 0.021 | | -0.0476 | 0.0292 | | 0.2444 | 0.1325 | | 0.0398 | 0.0452 | | -0.0492 | 0.0709 | |
| 2018 | 2018 | 0.2999 | 0.0482 | * | 0.5666 | 0.0574 | * | -0.3142 | 0.2884 | | 0.0698 | 0.0729 | | -0.0903 | 0.2174 | |
| 2018 | 2019 | 0.1537 | 0.0422 | * | 0.6209 | 0.0538 | * | 0.0586 | 0.1886 | | 0.2295 | 0.0519 | * | 0.1452 | 0.1132 | |
| 2019 | 2013 | 0.0001 | 0.0204 | | -0.0736 | 0.0364 | | -0.2333 | 0.154 | | -0.0094 | 0.0373 | | -0.1132 | 0.0618 | |
| 2019 | 2014 | -0.0046 | 0.0262 | | -0.0358 | 0.0253 | | 0.0915 | 0.1125 | | -0.0571 | 0.0298 | | 0.0536 | 0.0569 | |
| 2019 | 2015 | 0.0469 | 0.031 | | 0.0526 | 0.0301 | | 0.4068 | 0.1692 | | -0.0237 | 0.0323 | | -0.1575 | 0.0526 | * |
| 2019 | 2016 | -0.0287 | 0.027 | | -0.076 | 0.0373 | | 0.1722 | 0.1048 | | 0.1062 | 0.0171 | * | 0.1385 | 0.0512 | |
| 2019 | 2017 | -0.0256 | 0.0311 | | -0.1029 | 0.0363 | | 0.3678 | 0.1712 | | 0.0176 | 0.0506 | | 0.0743 | 0.0622 | |
| 2019 | 2018 | 0.0163 | 0.0355 | | 0.0271 | 0.0209 | | 0.0896 | 0.1372 | | 0.0495 | 0.0292 | | -3.8981 | 0.172 | * |
| 2019 | 2019 | 0.3367 | 0.0495 | * | 0.6214 | 0.0512 | * | 0.6301 | 0.1558 | * | 0.1134 | 0.0294 | * | 3.7183 | 0.2325 | * |

**Notes:** coefficients obtained after a Callaway-Sant'Anna DiD. The variables of the unique users per million affiliates, frequency per user, and expenditure per user are for technologies that had at least one user all years and are expressed in logarithms, therefore, coefficients can be interpreted as percentage changes. Standard errors in parentheses are computed with a bootstrap with 1,000 replications clustered at the technology level. Significant at 95% level: *.

Table F3: Impact of inclusion into HBP DiD CS: additional exercises

| | At least one user (1: Yes, 0:No) | | | Unique users per million affiliates | | | Expenditure per user (million COP) | | |
|---|---|---|---|---|---|---|---|---|---|
| **A. Only health procedures** | | | | | | | | | |
| Time | ATT | Std. Error | | ATT | Std. Error | | ATT | Std. Error | |
| -6 | 0.0221 | 0.0345 | | 0.3448 | 0.2326 | | -0.1498 | 0.1008 | |
| -5 | -0.0217 | 0.0418 | | -0.2884 | 0.159 | | 0.4266 | 0.1625 | * |
| -4 | -0.0121 | 0.0247 | | -0.2865 | 0.1729 | | -0.3508 | 0.1757 | |
| -3 | 0.0046 | 0.0194 | | -0.1219 | 0.0885 | | 0.0633 | 0.1164 | |
| -2 | -0.0293 | 0.0183 | | -0.1743 | 0.1285 | | 0.1342 | 0.1365 | |
| -1 | 0.0076 | 0.0168 | | 0.2396 | 0.1834 | | -0.3857 | 0.1385 | * |
| 0 ($1^{st}$) | 0.7191 | 0.0241 | * | 1.3704 | 0.1759 | * | 0.1992 | 0.1563 | |
| 1 ($2^{nd}$) | 0.5982 | 0.027 | * | 1.7081 | 0.2211 | * | 0.1123 | 0.0904 | |
| 2 ($3^{rd}$) | 0.5676 | 0.0296 | * | 1.8397 | 0.2163 | * | 0.1521 | 0.0988 | |
| 3 ($4^{th}$) | 0.589 | 0.0284 | * | 1.9999 | 0.2162 | * | 0.1037 | 0.122 | |
| 4 ($5^{th}$) | 0.5941 | 0.0476 | * | 2.1109 | 0.3184 | * | 0.0685 | 0.1576 | |
| 5 ($6^{th}$) | 0.6041 | 0.0481 | * | 2.3076 | 0.3459 | * | 0.024 | 0.1576 | |
| Overall | 0.612 | 0.0267 | * | 1.8894 | 0.2025 | * | 0.11 | 0.1075 | |
| **B. Only for technologies with HTA** | | | | | | | | | |
| | At least one user (1: Yes, 0:No) | | | Unique users (Thousands) | | | Expenditure per user (million COP) | | |
| Time | ATT | Std. Error | | ATT | Std. Error | | ATT | Std. Error | |
| -6 | 0.0221 | 0.0349 | | -0.3094 | 0.3052 | | -0.2261 | 0.1053 | |
| -5 | -0.0217 | 0.0423 | | -0.0816 | 0.0643 | | -0.1791 | 0.0581 | * |
| -4 | -0.0121 | 0.0246 | | 0.248 | 0.1175 | | 0.0086 | 0.0779 | |
| -3 | 0.0046 | 0.0191 | | 0.2493 | 0.1243 | | 0.006 | 0.055 | |
| -2 | -0.0293 | 0.0185 | | -0.0482 | 0.1188 | | -0.0153 | 0.0789 | |
| -1 | 0.0076 | 0.0163 | | 0.138 | 0.0798 | | -0.9033 | 0.1779 | * |
| 0 ($1^{st}$) | 0.7191 | 0.0243 | * | -0.234 | 0.2498 | | 0.5218 | 0.2175 | |
| 1 ($2^{nd}$) | 0.5982 | 0.0288 | * | -0.2224 | 0.2787 | | -0.2205 | 0.1028 | |
| 2 ($3^{rd}$) | 0.5676 | 0.0287 | * | 0.9425 | 0.4116 | | -0.1701 | 0.1608 | |
| 3 ($4^{th}$) | 0.589 | 0.0307 | * | 0.7068 | 0.4872 | | -0.3574 | 0.1571 | |
| 4 ($5^{th}$) | 0.5941 | 0.0476 | * | 0.1274 | 0.2647 | | -0.2735 | 0.1712 | |
| 5 ($6^{th}$) | 0.6041 | 0.0468 | * | 0.2758 | 0.2865 | | -0.3696 | 0.1847 | |
| Overall | 0.612 | 0.029 | * | 0.266 | 0.2514 | | -0.1449 | 0.1115 | |

**Notes:** coefficients obtained after a Callaway-Sant'Anna DiD. The variables of the unique users per million affiliates and expenditure per user are for technologies that had at least one user all years and are expressed in logarithms, therefore, coefficients can be interpreted as percentage changes. Standard errors in parentheses are computed with a bootstrap with 1,000 replications clustered at the technology level. Significant at 95% level: *.

Table F4: Impact of inclusion into HBP DiD CS: only for medications

| | At least one user (0 - 1 ) | | | Unique users per million affiliates | | | Expenditure per user (million COP) | | | IHH (0 - 10.000 ) | | | Institutional share (0 - 1 ) | |
|---|---|---|---|---|---|---|---|---|---|---|---|---|---|---|
| Time | ATT | Std. Error | | ATT | Std. Error | | ATT | Std. Error | | ATT | Std. Error | | ATT | Std. Error |
| -6 | 0.0363 | 0.0428 | | -0.1649 | 0.1716 | | -0.0251 | 0.0731 | | | | | | |
| -5 | 0.0263 | 0.0235 | | 0.2989 | 0.0908 | * | 0.1365 | 0.053 | | -1061.9022 | 408.6808 | | 0.0388 | 0.0422 |
| -4 | 0.0304 | 0.0333 | | 0.3771 | 0.1142 | * | 0.1687 | 0.0668 | | 129.5968 | 171.6554 | | -0.0238 | 0.0213 |
| -3 | -0.011 | 0.0194 | | -0.181 | 0.0919 | | 0.1601 | 0.0564 | * | 456.1577 | 188.9625 | | 0.0186 | 0.0198 |
| -2 | -0.1397 | 0.027 | * | -0.6233 | 0.1202 | * | 0.1692 | 0.0733 | | -59.4345 | 141.0786 | | -0.0255 | 0.016 |
| -1 | 0.0776 | 0.021 | * | -0.3141 | 0.0758 | * | -0.4923 | 0.0642 | * | 434.8642 | 156.9702 | * | 0.0136 | 0.0175 |
| 0 ($1^{st}$) | 0.0984 | 0.0211 | * | 1.5602 | 0.2773 | * | 1.3223 | 0.1942 | * | 93.585 | 129.2968 | | 0.007 | 0.0183 |
| 1 ($2^{nd}$) | 0.0785 | 0.026 | * | 1.5311 | 0.3106 | * | 0.414 | 0.2134 | | -31.1194 | 199.8306 | | 0.0237 | 0.0169 |
| 2 ($3^{rd}$) | 0.0662 | 0.0392 | | 2.5311 | 0.4245 | * | 1.1526 | 0.1843 | * | 39.356 | 196.3376 | | 0.0019 | 0.0313 |
| 3 ($4^{th}$) | 0.0518 | 0.0402 | | 2.1884 | 0.4622 | * | 1.0132 | 0.1746 | * | 135.7525 | 240.279 | | 0.0349 | 0.0211 |
| 4 ($5^{th}$) | -0.168 | 0.0154 | * | -1.7163 | 0.3074 | * | 2.9289 | 0.1762 | * | 1628.1765 | 532.7228 | * | -0.1912 | 0.0951 |
| 5 ($6^{th}$) | -0.177 | 0.0154 | * | -1.2595 | 0.3146 | * | 2.9328 | 0.1852 | * | 1517.7675 | 521.2388 | * | 0.0457 | 0.0533 |
| Overall | -0.0084 | 0.0199 | | 0.8058 | 0.2603 | * | 1.6273 | 0.1383 | * | 563.9197 | 227.8589 | * | -0.013 | 0.0308 |

**Notes:** coefficients obtained after a Callaway-Sant'Anna DiD. The variables of the Unique users per million affiliates and expenditure per user are for technologies that had at least one user all years and are expressed in logarithms, therefore, coefficients can be interpreted as percentage changes. Standard errors are computed with a bootstrap with 1,000 replications clustered at the technology level. Significant at 95% level: *.

# References


Abadie, A. (2021). Using synthetic controls: feasibility, data requirements, and methodological aspects. *Journal of Economic Literature*, *59*(2), 391–425. doi: 10.1257/jel.20191450

Abadie, A., Diamond, A., & Hainmueller, J. (2010). Synthetic control methods for comparative case studies: Estimating the effect of california's tobacco control program. *Journal of the American Statistical Association*, *105*(490), 493–505. doi: 10.1198/jasa.2009.ap08746

Abadie, A., & Gardeazabal, J. (2003). The economic costs of conflict: a case study of the basque country. *American Economic Review*, *93*(1), 113–132. doi: 10.1257/000282803321455188

Buitrago, G., Amaya-Nieto, J., Miller, G., & Vera-Hernández, M. (2023). Cost-sharing in medical care can increase adult mortality: evidence from colombia. *National Bureau of Economic Research Working Paper Series*, *No. 31908*. doi: 10.3386/w31908

Cavallo, E., Galiani, S., Noy, I., & Pantano, J. (2010). *Catastrophic natural disasters and economic growth* (IDB Working Paper Series No. IDB-WP-183). Washington D.C.. Retrieved from http://hdl.handle.net/10419/89155

Dirección de Regulación de la Operación del Aseguramiento en Salud, Riesgos Laborales y Pensiones (DROAS). (2020). *Selección de tecnologías para evaluar (priorización).* Bogotá, D.C.: Ministerio de Salud y Protección Social.

Escobar, M., Giedion, U., Giuffrida, A., & Glassman, A. (2009). Colombia: After a decade of health system reform. In A. Glassman, M. Escobar, A. Giuffrida, & U. Giedion (Eds.), *From few to many: ten years of health expansion in colombia* (pp. 1–14). Washington D.C.: Inter-American Development Bank.

Giedion, U., Panopoulou, G., & Gomez-Fraga, S. (2009). Diseño y ajuste de los planes explícitos de beneficios: el caso de Colombia y México. *Financiamiento del desarrollo*, *219*. Retrieved from https://repositorio.cepal.org/bitstream/handle/11362/5201/1/S0900746_es.pdf

Giedion, U., Tristao, I., Escobar, L., Bitrán, R., Cañón, O., Molins, S., ... Bolaño Prado, L. (2014). *Planes de beneficios en salud de América Latina: Una comparación regional.* Washington D.C.: Banco Interamericano de Desarrollo. Retrieved from https://publications.iadb.org/es/planes-de-beneficios-en-salud-de-america-latina-una-comparacion-regional

Hayati, R., Bastani, P., Kabir, M., Kavosi, Z., & Sobhani, G. (2018). Scoping literature review on the basic health benefit package and its determinant criteria. *Globalization and Health*, *14*(1), 26. doi: 10.1186/s12992-018-0345-x

Kluwer, W. (2021). *Uptodate.* Retrieved from www.uptodate.com

Leuven, E., & Sianesi, B. (2003). PSMATCH2: Stata module to perform full Mahalanobis and propensity score matching, common support graphing, and covariate imbalance testing. *Statistical Software Components, Boston College Department of Economics*.

Lozano, R., Fullman, N., Mumford, J., Knight, M., & et al. (2020). Measuring universal health coverage based on an index of effective coverage of health services in 204 countries and territories, 1990–2019: a systematic analysis for the Global Burden of Disease Study 2019. *The Lancet*, *396*(10258), 1250–1284. doi: 10.1016/S0140-6736(20)30750-9

Ministerio de Salud y Protección Social, Departamento Administrativo de Ciencia Tecnología e Innovación, Instituto de Evaluación Tecnológica en Salud (MinSalud, Colciencias, IETS). (2014a). *Guía de Práctica Clínica (GPC): Detección temprana y diagnóstico del episodio depresivo y trastorno depresivo recurrente en adultos. atención integral de los adultos con diagnóstico de episodio depresivo o trastorno depresivo recurrente* (Tech. Rep.). Bogotá, D.C.: Ministerio de Salud y Protección Social.

Ministerio de Salud y Protección Social, Departamento Administrativo de Ciencia Tecnología e Innovación, Instituto de Evaluación Tecnológica en Salud (MinSalud, Colciencias, IETS). (2014b). *Guía de Práctica Clínica (GPC) para el diagnóstico, tratamiento e inicio de la rehabilitación psicosocial de los adultos con esquizofrenia* (Tech. Rep.). Bogotá, D.C.: Ministerio de Salud y Protección Social.

Ministerio de Salud y Protección Social (MinSalud). (2014). *Calificación de criterios de priorización para inclusión de tecnologías en salud en la actualización del pos 2013: Propuesta técnica de tecnologías para inclusión al pos* (Tech. Rep.). Bogotá, D.C.: Ministerio de Salud y Protección Social.

Ministerio de Salud y Protección Social (MinSalud). (2020). *Selección de tecnologías para evaluar* (Tech. Rep.). Bogotá, D.C.: Ministerio de Salud y Protección Social.

Ministerio de Salud y Protección Social (MinSalud). (2021). *Pos pópuli.* Retrieved from https://pospopuli.minsalud.gov.co

Nuñez, J., Castañeda, C., Wiesner, D., Romero, J., Parra, J., Hurtado, C., ... Trujillo, A. (2015). *Evaluación de resultados e impacto de la unificación del Plan Obligatorio de Salud – POS, evaluación de*

*resultados de su más reciente actualización y evaluación de los procesos implementados en el marco de la unificacioń y actualización del POS*. Bogotá, D.C: DNP.

OECD. (2018). *Reviews of health systems: Colombia 2016*. Paris: OECD Publishing.

Unión Temporal: Econometría S.A. – SEI – SIGIL Consulting Group S.A. (Econometría, SEI, SIGIL). (2011). *Estudio de diagnóstico de la situación actual del acceso, uso racional y calidad de medicamentos, insumos y dispositivos médicos, que incluya la evaluación de la política farmacéutica nacional definida en el año 2003*. Bogotá, D.C.: Minsiterio de la Protección Social.

World Health Organization and others (WHO). (2007). *World health organization model list of essential medicines: 15th list* (Tech. Rep.).
